# Life histories of straight-tusked elephants from the Last Interglacial Neanderthal site of Neumark-Nord (~125 ka)

**DOI:** 10.1126/sciadv.adz0114

**Published:** 2026-03-13

**Authors:** Elena Armaroli, Federico Lugli, Théo Tacail, Lutz Kindler, Sabine Gaudzinski-Windheuser, Fulco Scherjon, Wil Roebroeks, Glendon Parker, Hubert Vonhof, Anna Cipriani, Thomas Tütken, Wolfgang Müller

**Affiliations:** ^1^ Department of Chemical and Geological Sciences, University of Modena and Reggio Emilia, 41125 Modena, Italy.; ^2^ Frankfurt Isotope and Elemental Research Center (FIERCE), Goethe-Universität Frankfurt, 60438 Frankfurt am Main, Germany.; ^3^ Institut für Geowissenschaften, Goethe-Universität Frankfurt, 60438 Frankfurt am Main, Germany.; ^4^ Division of Geological and Planetary Sciences, California Institute of Technology, Pasadena, CA 91125, USA.; ^5^ Institut für Geowissenschaften, Johannes Gutenberg–Universität Mainz, D-55128 Mainz, Germany.; ^6^ MONREPOS Archaeological Research Center and Museum for Human Behavioral Evolution, Leibniz Zentrum für Archäologie (LEIZA), Schloss Monrepos, 56567 Neuwied, Germany.; ^7^ Institute of Ancient Studies, Pre- and Protohistoric Archaeology, Johannes Gutenberg–Universität Mainz, 55122 Mainz, Germany.; ^8^ Faculty of Archaeology, Leiden University, P.O. Box 9514, 2300 RA Leiden, Netherlands.; ^9^ Department of Environmental Toxicology, University of California, Davis, CA 9516, USA.; ^10^ Department of Climate Geochemistry, Max Planck Institute for Chemistry, 55128 Mainz, Germany.; ^11^ Lamont-Doherty Earth Observatory, Columbia University, New York, NY 10964, USA.

## Abstract

Straight-tusked elephants (*Palaeoloxodon antiquus*) were the largest land mammals of the European Pleistocene. Abundant fossil remains have been recovered from Neumark-Nord [~125 thousand years ago (ka); Saxony-Anhalt, NE Germany], where over 70 individuals were accumulated by Neanderthal hunting and butchering activity. This study reconstructs the species’ paleoecology using a multiproxy approach that combines isotopic and paleoproteomic analyses of tooth enamel. LA-MC-ICPMS ^87^Sr/^86^Sr analyses on molar cross sections from four adults reveal subseasonal mobility over periods up to eight years. A specifically developed Sr isoscape facilitates tracking of elephant movements, using Bayesian analysis to map their Last Interglacial homerange. Amelogenin proteomic analysis identified three males and one likely female. Two male elephants exhibit elevated ^87^Sr/^86^Sr profiles, distinct from the local bioavailable ratios. Intra-tooth δ^13^C and δ^18^O analyses provide insights into paleodiet and drinking water sources, differing between individuals with low and elevated ^87^Sr/^86^Sr. The latter likely foraged up to 300 km away before arriving at Neumark-Nord, where they were ultimately killed and processed by Neanderthals.

## Teaser

Isotopes and proteomics reveal the mobility, diet, and sex of straight-tusked elephants hunted by Neanderthals at Neumark-Nord.

## INTRODUCTION

The straight-tusked elephant (*Palaeoloxodon antiquus*) was an iconic species of the European Pleistocene Interglacial ecosystem (*1*), sharing the landscape with Neanderthals during the warmer periods of the late Middle and Late Pleistocene. Its role as a resource for hominin populations has long been acknowledged, with archaeological findings demonstrating Neanderthal use of elephants as a source of food and their bones for tool making across Europe (*2*). Until recently, direct evidence that *P. antiquus* was actively hunted, rather than scavenged, remained scarce and debated (*3*–*5*). Similarly, our understanding of the behavioral ecology of these extinct elephants has remained limited and fragmented.

The lakeland of Neumark-Nord (near Halle, Saxony-Anhalt, NE Germany) offers a unique window into the ecology of *P. antiquus*, whose remains occur in exceptional abundance as a result of Neanderthal hunting activities during the Last Interglacial [Eemian, ~125 thousand years ago (ka)]. Neanderthals were almost continuously present around the water bodies for about 2000 years during the early part of the Eemian, and still, but less, active for another 2000 years (*6*, *7*). This unique and well-preserved archaeological and paleoenvironmental site complex has enabled multidisciplinary research, revealing substantial insights into Neanderthal adaptation to warmer, forested interglacial environments (*8*). Excavations at two synchronous lake-basin sites, Neumark-Nord 1 (NN-1) and Neumark-Nord 2 (NN-2), spanning about 26 hectares (Supplementary Text), uncovered rich assemblages of fossil animal remains, accumulated within a semi-open lake-dotted environment (*8*). The lakes attracted animals and thus provided Neanderthals with a continuous supply of prey, including species from both forest and open habitats (*6*, *9*). *P. antiquus*, considered to be a forest-dwelling species as it mostly occurs in interglacial settings, is represented by over 70 individuals, which forms the largest known fossil assemblage of this species worldwide (*6*). The cut mark distribution patterns on the bones indicate extensive exploitation of the carcasses by Neanderthals. The notable age and sex structure of the assemblage, skewed toward adult males (*10*, *11*), in combination with the rich cut mark record constitutes the earliest unambiguous evidence of selective *P. antiquus* hunting (*12*).

Genetic data from Neumark-Nord revealed that *P. antiquus* is closely related to the African forest elephant (*Loxodonta cyclotis*) (*13*, *14*). Reaching up to 4 m in shoulder height and weighing up to 13 tons (*15*), *P. antiquus* males were the largest herbivores of the Pleistocene, surpassing both extant male Asian and African elephants, as well as woolly mammoths (*16*). Given the availability of a range of smaller prey species in the landscape [e.g., horses, bovids, and cervids; (*9*)], likewise extensively hunted there, the decision to target these enormous animals raises questions about Neanderthal hunting strategies, group size, and potential methods of food storage. Estimates suggest that hunting, butchering, and processing of one of these elephants could have taken 3 to 5 days for a group of 25 individuals, providing enough food to last for months (approximately 2500 daily portions of 4000 kcal for adults, potentially feeding a group for at least 3 months) (*12*). This has led to speculations about Neanderthal food preservation techniques (e.g., drying and smoking), as well as the dynamics of Neanderthal local group size and mobility [see (*17*)]. It has recently been demonstrated that hunting of straight-tusked elephants was a wide-spread activity across Last Interglacial Europe, extending far beyond the case of Neumark-Nord (*18*). Given the importance of this species in Neanderthal subsistence strategies and our limited knowledge of this presumably forest-adapted elephant, further investigation into the ecology of *P. antiquus*, including its mobility patterns, its diet, and sex-related differences, is warranted and presented here.

Proboscidean remains found throughout the world have been widely studied using isotope analysis to reconstruct their diet (δ^13^C) and mobility (^87^Sr/^86^Sr, δ^18^O), as well as paleoenvironmental conditions (δ^13^C, δ^18^O, and δ^15^N), through both bulk and serial sampling of teeth and tusks (*4*, *19*–*22*). Sequential intra-tooth sampling has provided high resolution insights into their ecology during the several years it took for their molar teeth to completely form (*23*). However, most of the recent studies have focused on mammoths and mastodons [e.g., (*21*, *24*–*26*)], leaving *P. antiquus* ecology understudied and primarily inferred from stable isotope analysis [see (*27*)] or comparison with modern proboscidean species. Roditi *et al*. (*27*) recently conducted the first intra-tooth multi-isotope analysis on a *P. antiquus* molar from a Middle Pleistocene (MIS 12) site in Greece, but their spatial resolution did not match the high temporal resolution of Kowalik *et al*.’s (*21*) work on woolly mammoth mobility. Similar studies on *P. antiquus* for the Last Interglacial are still lacking, as is a comprehensive analysis combining isotope and proteomic data on tooth enamel to explore potential sex-specific behavior in mobility and feeding.

This study aims to provide a more comprehensive picture of *P. antiquus* ecology and habitat use during the Last Interglacial. We present the first multiproxy analysis including isotope and amelogenin analysis of the last Interglacial straight-tusked elephant molars (i.e., enamel). Using in situ laser ablation (LA) multicollector inductively coupled plasma mass spectrometry (MC-ICPMS), we performed ^87^Sr/^86^Sr analysis on four halved *P. antiquus* molars from both NN-1 and NN-2 sites to reconstruct movement behavior at subseasonal resolution. Two of the teeth were recovered from autochthonous kill sites in the lower shore horizons at NN-1 (*18*), while the other two originated from the allochthonous thanatocoenosis of the “fat factory,” where the remains had been transported to for bone processing at the pond margin of NN-2 (*28*). Our results are interpreted using a Sr isoscape of NE Germany specifically built for this research. Sequential intra-tooth δ^13^C and δ^18^O isotope analyses and sex determination by amelogenin (AMELX or AMELY) were performed by isotope ratio mass spectrometry (MS) and liquid chromatography–tandem MS (LC-MS/MS), respectively, to link any changes in mobility to environmental seasonality and assess potential behavioral differences between sexes.

## RESULTS

**Fig. 1.**
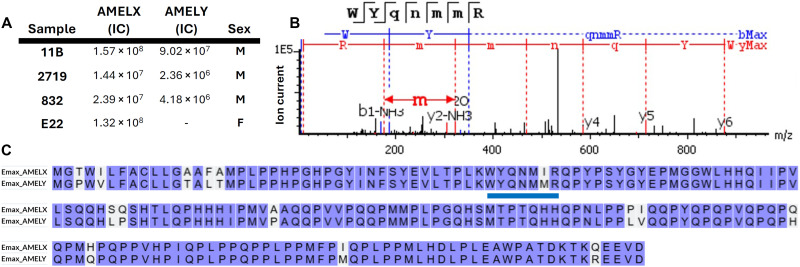
LC-MS/MS proteomic results and sex estimation. Amelogenin (X and Y) amino acid sequences were translated from the *Elephas maximus* genome and aligning peptides were quantified in PEAKS Xpro and used to estimate male (XY) and female (XX) sex. (**A**) The cumulative ion current (IC) from both amelogenin genes was quantified for each *P. antiquus* individual. (**B**) An unambiguous example of the AMELY-specific double methionine motif in the WYQNMMR peptide form individual 11B is represented. Transitions from methionine 46 are labeled. (**C**) Two paralogs of the *E. maximus* amelogenin gene product (Emax_AMELX, Emax_AMELY) were aligned in UNIPROT. Sex chromosome specific amino acids are indicated along with the peptide sequence for the spectrum in (B) (blue bar).

Three of four individuals show the presence of both AMELY and AMELX sequences, strongly suggesting male sex (Fig. 1). Individual E22, despite the high-AMELX ion current (Fig. 1) lacks unique AMELY peptides, indicating likely female sex. Other endogenous dental proteins were identified through MaxQuant searches, namely, enamelin, ameloblastin, and matrix-metallopeptidase 20.

The two molars from the NN-1 site yielded average enamel ^87^Sr/^86^Sr values of 0.70965 (11B) and 0.70953 (E22) (0.0005 and 0.0003 1SD, respectively), with values ranging between 0.70883 and 0.71125 (11B) and between 0.70887 and 0.71065 (E22). In contrast, the mean enamel ^87^Sr/^86^Sr values of the two molars from the NN-2 site are 0.71377 (832A) and 0.71375 (2719A) and thus much higher, which is also reflected in their two to three times larger 1SD values of 0.001 and 0.0008, respectively. The ^87^Sr/^86^Sr values range between 0.71136 and 0.71631 (832A) and between 0.71163 and 0.71631 (2719A) (see fig. S1 for the Sr isotope profiles). These profiles represent an ontogenetic range of 8 to 10 years, as we obtained a modeled molar formation age of 8 years (*23*) for sample E22 (fig. S3). LA Sr isotope profile data are reported in table S2.

**Fig. 2.**
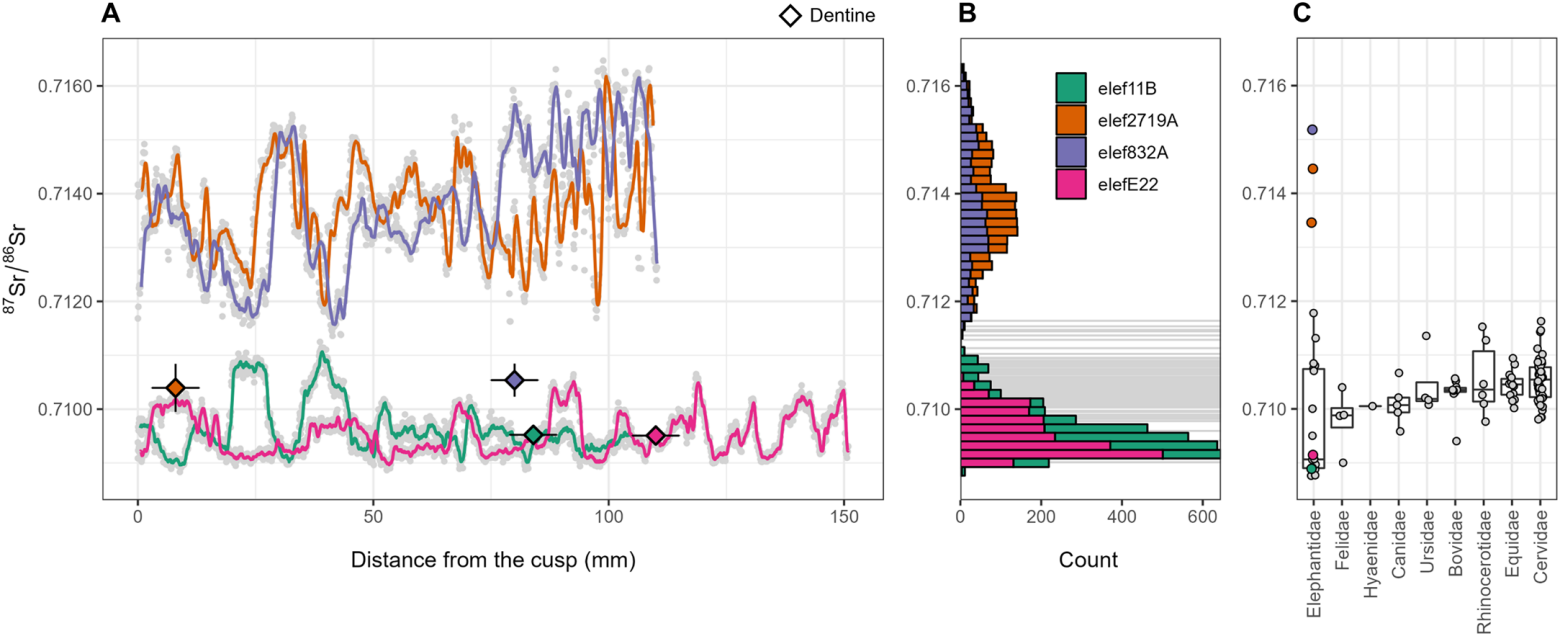
^87^Sr/^86^Sr profiles of *P. antiquus* tooth enamel and comparative bulk enamel data of other large mammals from Neumark-Nord. (**A**) The enamel intra-tooth ^87^Sr/^86^Sr profiles, measured in growth-direction by LA-MC-ICPMS, of the four individuals are plotted against distance from the cusp. The orange and purple profiles correspond to the more radiogenic 2719A and 832A individuals from NN-2, while the less radiogenic 11B and E22 individuals from NN-1 are represented by the green and pink profiles. Diamonds represent dentine values; vertical error bars are 2SD, while horizontal bars are the approximate line length. (**B**) Histogram plots compare the variability and the distribution of ^87^Sr/^86^Sr values for each profile; light gray bars are ^87^Sr/^86^Sr values obtained from bulk enamel samples of different, near-contemporaneous mammals at Neumark-Nord (elephants excluded), with the local baseline likely lying within this range. (**C**) Box plots representing bulk enamel ^87^Sr/^86^Sr of the Neumark-Nord fauna; enamel values of elephants measured with solution mode MC-ICPMS are colored as the profiles; ID 2791A has been sampled and measured two times with solution MC-ICPMS.

The faunal bulk enamel samples used to assess the local bioavailable Sr revealed an overall median ^87^Sr/^86^Sr value of 0.7104 (Fig. 2 and table S3). To assess the ^87^Sr/^86^Sr value of the local diagenetic endmember, one LA-MC-ICPMS line per tooth was measured on dentine (which is more prone for postmortem Sr alteration), yielding the following results: 11B shows an ^87^Sr/^86^Sr of 0.7095 ± 0.0001 (1SD), E22 of 0.7095 ± 0.0001, 832A of 0.7105 ± 0.0002, and 2719A of 0.7104 ± 0.0002. As expected for diagenetically altered specimens, these values tend toward the local baseline range. Yet, part of the original biogenic Sr isotope composition is perhaps still preserved in the dentine; indeed, individuals 832A and 2719A show the more radiogenic values, still partly reflecting their higher enamel values compared to 11B and E22 (fig. S3). Overall, this suggests that the ^87^Sr/^86^Sr of dentine is at least partially altered by postdepositional uptake of local, sediment-derived Sr, while enamel probably still preserves biogenic ^87^Sr/^86^Sr. This evidence is also consistent with the large intra-tooth ^87^Sr/^86^Sr fluctuations observed within the enamel LA lines: a strong and diffuse diagenetic alteration would have homogenized the Sr isotope composition [see, for example, (*29*)]. The summary list of all isotopic and proteomic results can be found in the Supplementary Materials (table S4).

**Fig. 3.**
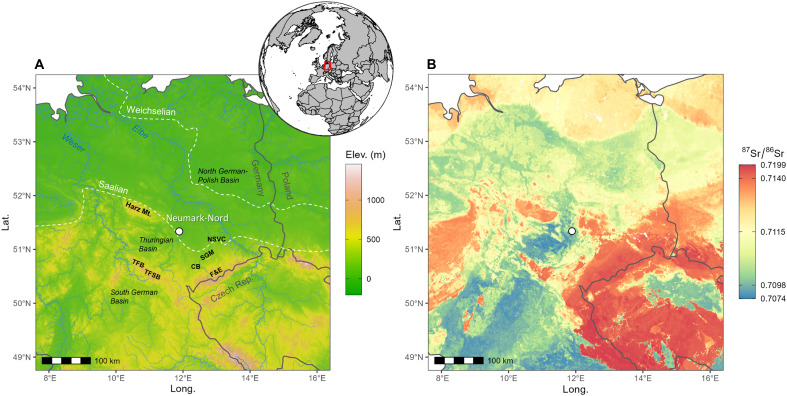
Geographical setting and Sr isoscape of NE Germany. (**A**) The Digital Elevation Model was obtained from the get_elev_raster R function (https://github.com/USEPA/elevatr), which uses the Amazon web Services (https://registry.opendata.aws/terrain-tiles/) terrain tiles and the Open Topography Global Datasets API. Rivers are from (*30*). The white dashed lines represent the maximum glacial extents during the Saalian and Weichselian periods, which occurred before and after the Eemian interglacial (as represented by the Neumark samples investigated), respectively. F&E, Fichtelgebirge and Erzgebirge mountains; TFB, Thuringian Forest Basin; TFSB, Thuringian Franconian Slate Belt; NSVC, North Saxon Volcanic Complex; SGM, Saxon Granulite Mountains; CB: Chemnitz Basin. (**B**) The isoscape was developed using the randomForest package with ecogeological external predictors (*n* = 23) [see (*96*, *100*)]. The isotope map is cropped at approximately 300 km around the Neumark-Nord site (white circle), with a median ^87^Sr/^86^Sr value of 0.7104. The modeled ^87^Sr/^86^Sr of the area (longitude: 8–16°E and latitude: 49–54°N) range between 0.7074 and 0.7199. The maps were made with R (version 4.0.5).

Neumark-Nord is located on Cenozoic sediments of the North German Basin (*30*), characterized by relatively low ^87^Sr/^86^Sr values (see fig. S4). More radiogenic bedrock areas are present all around the site (Fig. 3) and mainly formed by Precambrian and Paleozoic granitic gneisses, granites, rhyolites, and siliciclastic sediments (*30*). These areas are characterized by ^87^Sr/^86^Sr values > 0.7120 (Fig. 3), thus being possible areas of origin of the more radiogenic elephant samples (i.e., 832A and 2791A).

**Fig. 4.**
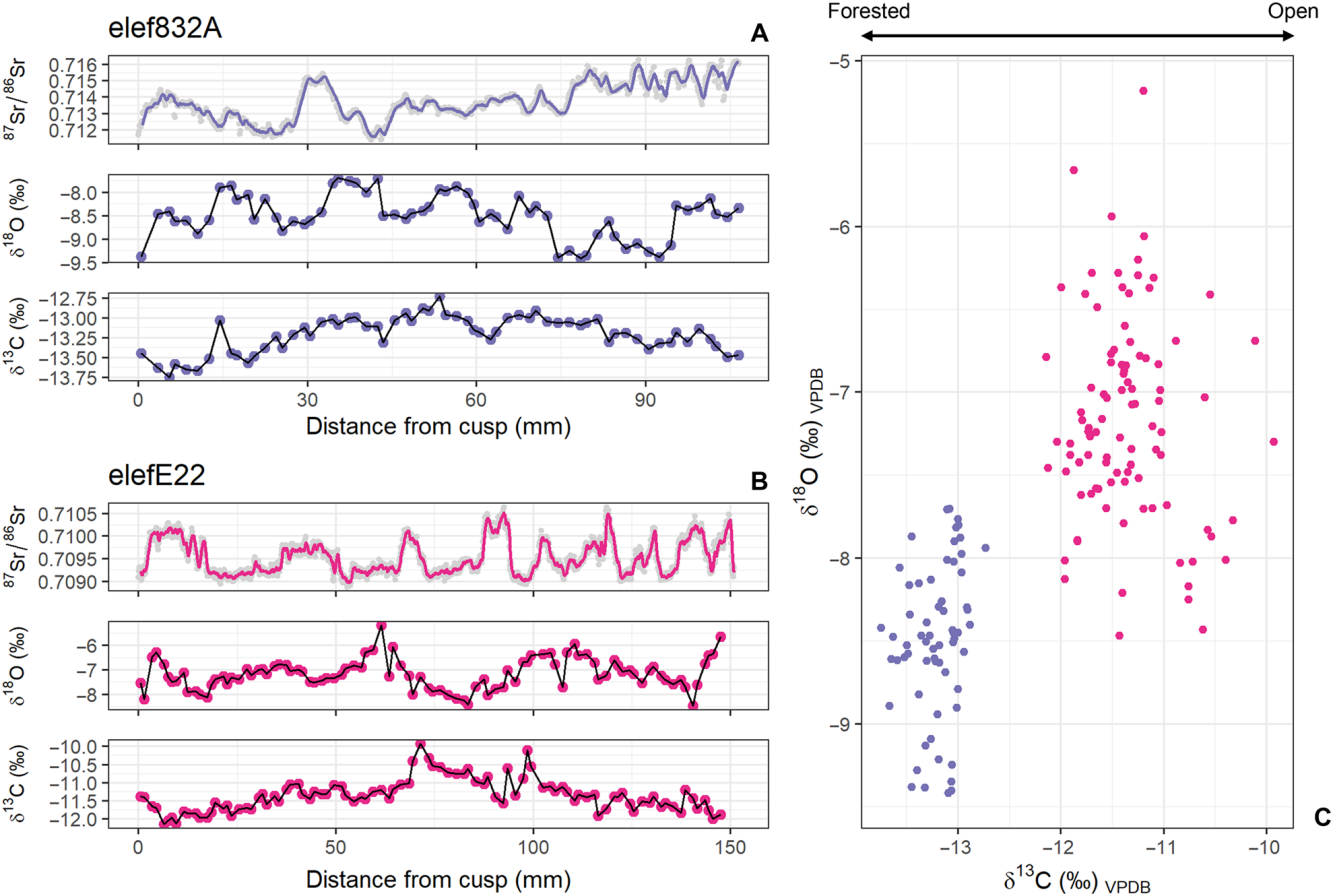
Comparison of spatially-resolved enamel strontium, carbon and oxygen isotope profiles. (**A** and **B**) δ^18^O_VPDB_ and δ^13^C_VPDB_ (‰) and ^87^Sr/^86^Sr of 832A (A) and E22 (B). (**C**) In the cross plot of δ^18^O_VPDB_ and δ^13^C_VPDB_ (‰) each symbol represents one of the serial enamel samples drilled along the crown length. The two individuals form two nonoverlapping clusters, with 832A (purple) showing more negative values, likely linked to a more forested habitat and ingestion of more ^18^O-depleted water.

The individual 832A yielded δ^13^C_VPDB_ values between −13.7 and −12.7‰ (mean = −13.2‰, 1SD = 0.2, and *n* = 68) and δ^18^O_VPDB_ values between −9.4 and −7.7‰ (mean = −8.5‰, 1SD = 0.5, and *n* = 68). The less radiogenic E22 sample showed δ^13^C_VPDB_ values ranging from −12.1 and −9.9‰ (mean = −11.4‰, 1SD = 0.4, and *n* = 96) and δ^18^O_VPDB_ values from −8.5 and −5.2‰ (mean = −7.2‰, 1SD = 0.6, and *n* = 96). The two individuals (both represented by an upper M5) form two distinct, nonoverlapping clusters, with the more radiogenic sample 832A showing more negative δ^13^C and δ^18^O values (Fig. 4). On the other hand, the less radiogenic sample E22 shows higher intra-tooth variability (2.2 and 3.4‰ versus 1 and 2.2‰ for δ^13^C and δ^18^O, respectively).

Intra-tooth δ^18^O values and ranges (∼3‰) of both samples are in line with those of *P. antiquus* from other German Interglacial sites (*31*) and provide information on seasonal variations in local environmental conditions (*32*). The highest and lowest δ^18^O values correspond to the warmer and the colder months of the year, respectively, as typically seen in temperate environments of mid- and high latitudes (*33*). Sequential δ^13^C values of both individuals are typical of C_3_ plant feeders, in line with European plant distribution (*34*) and what we know from extant and fossil proboscideans in western Europe (*35*). The presence of a C_3_-plant-dominated environment has been further confirmed by δ^13^C values of bovid and horse bone collagen from the NN-2 site (*36*).

## DISCUSSION

### Mobility

Addressing the mobility behavior of extinct proboscideans is challenging, but isotopic analyses are increasingly used to unravel this complex phenomenon (*4*, *21*, *26*). In the case of *P. antiquus*, only a few studies are available (*27*) and much of our understanding still relies on comparison with extant elephants. Modern elephants exhibit highly plastic mobility behavior, in both Africa and Asia, with documented patterns that include migration (i.e., movements between nonoverlapping ranges), nomadism (i.e., irregular movement patterns), and ranging (i.e., movements within one home range or within and between two overlapping home ranges) (*26*). The type of movement varies with environment, climate, and nutritional requirements, but also with social behavior and personality. Differences in mobility are also influenced by sex as well as inter- and intra-individual (year-to-year) differences have been observed (*26*, *37*, *38*). Based on ecological studies, modern elephants can have very large seasonal and annual home ranges between 37 and 32000 km^2^ for African savanna elephants and 37 to 2250 km^2^ for African forest elephants, respectively (*26*). One-way travel distances during migration in *Loxodonta africana* range from 20 to 300 km, and may extend up to 700 km (*39*, *40*). Although we cannot state with any certainty that extinct *P. antiquus* had home ranges and migration distances similar to those of modern elephants, the size of our cropped isoscape map of NE Germany (Fig. 3) was informed by such modern analogue studies.

**Fig. 5.**
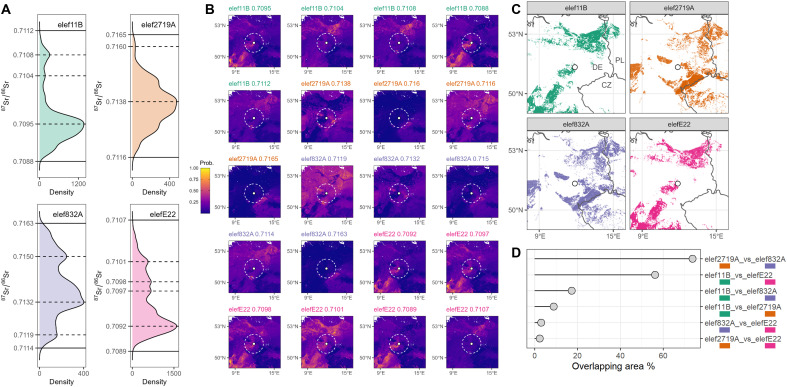
Geographic assignment of Neumark-Nord *P. antiquus*. (**A**) Calculated density functions from LA-MC-ICPMS ^87^Sr/^86^Sr data; modal values (dashed lines) were calculated for each profile to evaluate the most common ^87^Sr/^86^Sr ratios within LA profiles: These values, together with maximum and minimum (solid lines), were then used for the Bayesian provenance approach against the Sr isoscape. (**B**) Provenance probabilities are estimated through a Bayesian approach, with 0 (blue) representing low-probability areas and 1 (yellow) high-probability areas; the white spot marks the site of Neumark-Nord. The white dashed circle marks a 100 km radius around the site. Maximum, minimum, and modal ^87^Sr/^86^Sr values (A) were tested for each individual. The geographical framework is the same as Fig. 3. (**C**) All the top 5% probability maps for each elephant (see fig. S5) were joined and plotted together, showing partial overlaps of the overall residence areas. (**D**) A relative overlap (%) of the areas for elephant couples was calculated.

Our geographical provenance modeling (Fig. 5) indicates almost nonoverlapping areas for the two elephant groups, i.e., those with low versus high ^87^Sr/^86^Sr, suggesting remarkably different home ranges. Individuals 832A and 2179A, showing the highest ^87^Sr/^86^Sr values of the dataset (mean = 0.71377 and 0.71375, respectively), are compatible with highly radiogenic Precambrian to Paleozoic bedrock areas located within 300 km radius of the site (i.e., with ^87^Sr/^86^Sr > 0.7120 to 0.7130; Figs. 3 and 5).

One of these areas with old crustal bed rocks is located NW of Neumark-Nord and represented by the Harz Mountains (<100 km), consisting of Precambrian to Early Paleozoic marine sedimentary rock such as greywackes and phyllites that are slightly metamorphosed and intruded by some Late Paleozoic granites and rhyolites. Equally radiogenic ^87^Sr/^86^Sr can also be encountered NE of the site, in an area corresponding to the North Saxon Volcanic Complex (<50 km), formed by Late Carboniferous to Early Permian volcaniclastic, subvolcanic, and epiclastic rocks. In the SE of the site larger areas with radiogenic metamorphic and magmatic bedrocks are also present, corresponding to the Saxon Granulite Mountains composed of granulites surrounded by metamorphosed phyllites, quartzites, graywackes, and shales; the Chemnitz Basin mainly formed by Late Carboniferous to Early Permian terrestrial conglomerates and sandstones, with intercalated rhyolites, as well as the Fichtelgebirge and Erzgebirge mountains (~100 km) formed mainly by Precambrian to Paleozoic clastic metasedimentary and igneous rocks with a few Late Paleozoic granites and rhyolites. To the SW of the site, next to the Fichtelgebirge and Erzgebirge mountains, there are the Thuringian-Franconian-Slate Belt and the Thuringian Forest Basin formed by Triassic terrestrial siliciclastic rocks and marine limestones (~100+ km) [see figure 1 (b and c) in (*30*)]. Although there is some evidence that elephants avoid climbing steep slopes because of energy cost, injury risk, and lack of forage (*41*), their presence have been attested in certain African (*42*) and Asian mountainous regions, typically at relatively low elevations [<1500 m; (*43*)], although they have occasionally been recorded at altitudes as high as 3250 m (*44*). Given the Eemian climate optimum and wide forest expansion documented in Europe during the Last Interglacial (*45*–*47*), all these relatively low elevation areas (<1300 m) with more radiogenic ^87^Sr/^86^Sr than in the vicinity of Neumark-Nord should have been accessible to *P. antiquus* and thus represent the most likely places of origin for these elephants with high ^87^Sr/^86^Sr in their molars. However, it cannot be ruled out that glacial transport, particularly during the Saalian glaciation (see Fig. 3 for maximum ice-sheet extension), may have transported detritus of old crustal rocks with highly radiogenic Sr isotope signatures from regions such as Scandinavia (*48*) to areas in the vicinity of Neumark. If such moraines covering the local bedrocks would be the source for the bioavailable Sr, then *P. antiquus* could also have incorporated high ^87^Sr/^86^Sr from these Pleistocene glacial sediments.

Based on the highest and the lowest ^87^Sr/^86^Sr values of their intra-tooth profiles, we cannot exclude that individuals E22 and 11B dwelled in low-radiogenic areas SW and NE of the site, still within approximately 300 km of distance (Fig. 3). However, considering (i) the modal ^87^Sr/^86^Sr values of the two samples (Fig. 5), (ii) the similar ^87^Sr/^86^Sr values compared to other mammal taxa used for baseline assessment (Fig. 2), and (iii) the comparison with the only existing study on *P. antiquus* mobility, which indicates a high degree of site fidelity in this species (*27*), it is most parsimonious to assume that the home range of these two individuals was in vicinity of the site.

Our inferences on *P. antiquus* mobility rely on the isotope values along the profiles, specifically the modal values and the observed maxima and minima. We cannot, however, ultimately determine whether these values fully equilibrated with their respective environmental Sr endmembers. Evidence from large mammals indicates that Sr pools with long turnover times can contribute to dental Sr isotope compositions, potentially damping temporal variability in the resulting time series [e.g., (*19*)]. At the same time, fast-turnover Sr pools are also present, generating rapid shifts in Sr isotope ratios while simultaneously overprinting long-term mixing trends (*19*, *49*). Recent high-spatial-resolution analyses of mammoth enamel (*49*) reveal clear subannual Sr-isotope variability, which argues against substantial (>months) Sr pooling within proboscidean bodies. Overall, to minimize the confounding effects of enamel maturation and long-term Sr mixing, analyses should be conducted along the enamel-dentine junction, where the enamel maturation overprint is minimal (*50*).

### *P. antiquus* habitat and ecology

In Northern Europe the Last Interglacial is subdivided into different pollen assemblage zones (PAZs) that span approximately 11,000 years and reflect the vegetation succession after the preceding Saalian glacial period. At Neumark-Nord, Neanderthals were present throughout the first part of the Eemian Interglacial. Their continuous presence of approximately 2500 years has been indeed documented from PAZ II/III (*Pinus/Quercus* phase) to PAZ IVb (*Corylus* phase), with sporadic attestations during PAZ V (*Carpinus* phase) (*6*, *12*). Apart from animal taxa found within the archaeological sequence (*9*), the presence of a semi-open environment around the Neumark-Nord paleolakes during these stages is demonstrated by the pollen record, where upland herbs were documented along with tree taxa, especially in the PAZs III and IV (*51*). The relative openness of the environment was probably the result of a combination of factors, including Neanderthal use of fire (*6*), local climate and herbivore activity (i.e., feeding, trampling) around the lake basin, which in this period was characterized by an increased water supply. A more closed forest environment is documented from PAZ V onward, when the evidence of Neanderthal presence starts to disappear. These data overall suggest Neanderthal preference for semi-open environments (*52*, *53*), which they may have helped to create through their use of fire in this landscape (*6*). The carbon isotope results (Fig. 4) on *P. antiquus* enamel conducted in this study further support this palynology-based environmental reconstruction.

The usefulness of δ^13^C analysis in C_3_-dominated environments is well established, and helps with the distinction of different ecological niches (*54*, *55*). Studies of a variety of plant remains from the Neumark-Nord sites have shown the presence of substantial open areas around the lakes, with more wooded, C_3_-dominated environments at some distance, minimally 1 km from the sample sites (*6*, *52*), in line with recent work emphasizing the heterogeneity of Last Interglacial vegetation [see, e.g., (*56*)]. Our δ^13^C results confirm different feeding behavior, and consequently different habitats, between the likely nonlocal 832A and local E22 individuals. While the δ^13^C values of the latter are typical of semi-open environments (*57*), the on average ~2‰ more negative values of the former are likely indicative of the so-called “Canopy effect,” reflecting feeding in a more forested environment characterized by ^13^C-depleted subcanopy plants (*58*, *59*). Furthermore, our results agree with ecological data on fossil and modern proboscideans. Extant savanna and forest elephants are indiscriminate feeders, with a diet based on grass, fruit and different parts of trees and shrubs, depending on what the surrounding environment has to offer. The same is generally supposed for *P. antiquus*, living in open woodlands of temperate ecosystems (*26*, *60*), and confirmed by the gut content from two *P. antiquus* individuals found in NN-1 containing a variety of leaves, twigs from different tree species, grasses, herbs, and shrubs (*61*, *62*). Its dietary flexibility has been demonstrated through microwear and mesowear patterns of tooth enamel, with the latter also documenting seasonal variations in proboscidean diet (*63*–*65*). Overall, our δ^13^C results are comparable to those previously obtained from *P. antiquus* from NN-1 (*61*) and consistent with findings for the same species in Italy (*66*) and Germany (*31*).

δ^18^O values of mammalian enamel hydroxylapatite reflect those of body water, which in turn are influenced by multiple factors, including temperature, precipitation, and animal feeding and drinking behavior (*67*). Elephants ingest one third of their water needs from plant material (*60*). However, as obligate drinkers (*41*), their body water δ^18^O values are strongly influenced by local meteoric water, although deviations may occur due to the contribution of large rivers or other surface water sources (*67*). During the first part of the Eemian Interglacial, continental climate characterized by warm temperatures, relatively cold winters and low precipitation rate has been widely documented in Northern Europe (*47*, *68*, *69*), even though short climatic oscillations could have been more frequent than commonly thought (*45*). Both individuals analyzed in this study showed δ^18^O values compatible with those obtained for *P. antiquus* from other German Interglacial sites (*31*). However, higher δ^18^O values have been reported for other *P. antiquus* specimens from the NN-1 site, interpreted as a combination of warm climate and evaporative enrichment in the lake (*32*, *61*). As for δ^13^C values, individual 832A showed more negative δ^18^O values than E22 (Fig. 4). This difference is likely due to this individual’s feeding and drinking in a different, more forested and thus humid environment. Moreover, it could potentially be related to an altitudinal effect on both precipitation and temperature δ^18^O values, which might support the hypothesis that individual 832A originated from, or spent time in, nearby mountainous areas before moving to Neumark-Nord. In addition, differences in hydrological settings of these regions–such as the presence of rivers transporting water from distinct catchments–could have further influenced δ^18^O values (*67*). When C and O isotope results are coupled with ^87^Sr/^86^Sr values (Fig. 4) it becomes particularly evident that the two individuals roamed in two different areas, characterized by different geological bedrock substrates. Consequently, all the isotope values clearly reflect the differences in food and water ingested in different habitats over multiple years.

Both individuals display intra-tooth C and O isotope fluctuations. The only exception is the low (<1‰) intra-tooth δ^13^C variation of 832A. However, the dampening influence during enamel mineralization that reduces the C-O isotope signal variability in tooth enamel must be considered (*70*, *71*). The long maturation time during the amelogenesis causes an attenuation of the δ^13^C and δ^18^O isotope signal, leading to a dampening and thus biasing of past seasonality reconstructions (*19*, *27*, *71*, *72*). In proboscidean, dampened enamel signals may capture as little as ~40% of the original water or food C-O isotopic variability (*72*). Therefore, it is reasonable to expect that our isotope data are affected by comparable dampening effects. An additional dampening effect for δ^18^O values could have been caused by drinking from large water bodies such as lakes, less representative of seasonally varying local precipitation values due to mixing of water from different catchment areas (*67*). Last, the observed, albeit limited, δ^13^C variations could reflect differences in the carbon isotope composition of the plants consumed by the elephants, including both variation between plant species and between different parts of the same plants (*55*, *66*, *73*).

Diet seasonality is widely documented in extant species and depends on both seasonal variation in resources and elephant movement behaviors, two factors strongly linked to each other (*74*, *75*). However, the δ^13^C and δ^18^O intra-tooth values of both individuals E22 and 832A do not seem to be correlated, or are at best only slightly correlated, to intra-tooth ^87^Sr/^86^Sr changes (*R^2^* = 0.008 and 0.175, respectively; fig. S6). Therefore, although elephants’ nature of being obligate drinkers and foraging activities strongly affects their movement behavior (*41*), often driven by seasonal changes in rainfall and food availability (*76*–*78*), it is not possible to state with certainty that the Sr isotope variations detected in our samples (i.e., their mobility) are directly linked to seasonal climatic fluctuations. However, we know from modern ecological studies that individuals who migrate within a population do not necessarily do so every year or on a seasonal basis (*26*, *39*). In addition, intrinsic methodological limitations in sampling for O and C isotope analyses impose a lower spatial (and thus temporal) resolution compared to laser-based Sr isotope analysis, hampering a direct one-to-one correlation of the two proxies. Overall, it is likely that the intra-tooth stable isotope signal results from the mix of subannual climatic fluctuations in the local environment and the elephants’ mobility. Yet, a cross-correlation analysis (fig. S8) for individual 832A reveals a negative autocorrelation (*r* < −0.5) of δ^18^O and ^87^Sr/^86^Sr at a lag of −2 months. Cautiously, this may suggest a delay of about 2 to 4 months in mobility (Sr) relative to seasonal change (O).

Combining Sr-C-O isotope analysis of enamel clearly identifies two individuals with different mobility patterns that are roaming for several years in distinct geological areas characterized by different vegetation (including canopy cover) and water sources. It seems that during the ~8 years of life represented by the molars analyzed the two individuals never roamed in the same area as their isotopic values do not overlap. The individual 832A with more negative δ^13^C values and more radiogenic Sr in its enamel likely dwelled in one of the mountainous areas present within 300 km of Neumark-Nord characterized by elevated ^87^Sr/^86^Sr values (Figs. 3 and 5).

While primarily roaming in forests, extant African forest elephants are known to spend one-third of their time in forest clearings, locally known as “bais,” especially during the wet season (*79*). These clearings, characterized by herbaceous vegetation, water sources, and minerals, play a crucial role in meeting elephants’ nutritional needs. Through their activities, elephants contribute to the formation and maintenance of bais, which also serve as important social gathering sites (*80*, *81*). The Neumark-Nord lakes clearly attracted a wide range of herbivores during the first part of the Eemian Interglacial, including *P. antiquus*. Most individuals (MNI = 52) were retrieved from lower littoral zone deposits that accumulated over an estimated period of 300 years (*12*). Since only a portion of the fauna once accumulated around the lakes could be recovered (*12*), we can infer that Neumark-Nord likewise served as a point of attraction, and possibly aggregation, for *P. antiquus*. Neanderthals may have taken advantage of this to hunt *P. antiquus*, possibly contributing to the maintenance of the landscape’s openness through fire use (*6*).

The most parsimonious and plausible explanation is that the two most radiogenic adult male individuals arrived in the Neumark-Nord landscape at a time after the complete mineralization of the analyzed molar teeth. This is in line with the largely exploratory behavior documented in extant elephants in response to food availability, mating opportunities, and short-term environmental changes (*79*, *81*, *82*). This interpretation is also plausible based on the known distance (up to 700 km) traveled by extant and fossil proboscideans during migration (*40*) or other types of movement (*26*). In addition, it is in line with the current interpretation of Neumark-Nord as an area where Neanderthals were active in all seasons, with an almost continuous presence of resources possibly allowing for a less mobile lifestyle than commonly acknowledged (*9*, *12*).

Within this ecological and behavioral context, this study provides the first comprehensive characterization of the ecology of *P. antiquus* during the Last Interglacial. Moreover, it demonstrates that Neanderthals hunted both male and female individuals from different geographically separated elephant populations that migrated to and gathered around the lake basins of Neumark-Nord. Dental enamel proteomics reveals that the four individuals studied were three males and one possible female. We found that all individuals were seasonally mobile. However, while based on their Sr isotope composition two individuals, one male and one probably female, likely roamed for years not far from Neumark-Nord, the other two (males) dwelled in areas with more radiogenic bioavailable Sr, compatible with mountainous regions within 300 km of the site. Through serial intra-tooth C and O isotope analysis, we confirmed the semi-open nature of the landscape in the vicinity of Neumark-Nord. By combining Sr-C-O isotope data from the more radiogenic 832A and less radiogenic E22 individuals, we found evidence that they roamed in distinct geological areas characterized by different types of vegetation (more closed versus more open forests, respectively), and possibly different elevation and/or hydrology.

While the number of animals investigated here is relatively low, the results are in line with observations of differences in the spatial behavior of extant male and female elephants, with males generally being more mobile and traveling longer distances, unconstrained by the need to stay with a group, in contrast to females. Our biogeochemical analyses support the inference that the focus on adult males at Neumark-Nord was driven not only by their larger size, and therefore greater yield of edible resources, but also by their more solitary lifestyle, which likely made them easier to hunt than females (*12*).

This multiproxy approach combining enamel amelogenin, time-resolved isotope analysis, and isoscape provenance probability of mammal teeth could become a blueprint for a standard practice in future archaeological and paleontological life history reconstructions of both animals and humans. It provides a powerful toolbox for gaining unprecedented insights into the behavioral ecology of extinct species, including sex-specific differences.

## MATERIALS AND METHODS

### Dental specimens

Based on their distinct bulk enamel Sr isotope compositions, four molars (M4 to M6) of *P. antiquus* [age classes XIV to XXVI sensu (*83*), i.e., 16 to 47 years old] from Neumark-Nord (Supplementary Text and fig. S7) were selected for spatially resolved strontium isotope (^87^Sr/^86^Sr), proteomic (AMELX and AMELY), and serial stable isotope analyses (δ^13^C and δ^18^O) of tooth enamel along the tooth growth axis. Two molars (samples 11B and E22) came from different layers of the NN-1 site [NN1.4 lower gyttja and NN1.6.1 lower littoral, PAZ I–III with a duration of 750 years and the subsequent early phase of PAZ IVa with a duration of 1200 years, respectively; (*9*)]. The other two molars (samples 832A and 2719A) came from the same layer in basin NN2 belonging to a later phase PAZ IVa [NN2/2B; weighted mean age 121 ± 5 ka, (*6*, *9*)] (see the Supplementary Text).

### Enamel proteomics

For each tooth, a small enamel fragment (approximately 200 mg) was drilled and used for amelogenin-based sex determination by LC-MS/MS. This technique exploits the sexual dimorphism of AMELX and AMELY protein isoforms, expressed from X and Y chromosomes, with the latter being present in the dental enamel proteome of males only [see (*84*–*86*)]. Enamel powder was digested with 250 μl of 1.2 M HCl to extract peptides. Acid extracts were purified with in-house C18 STAGE tips, and peptides eluted with 60% acetonitrile in 0.1% formic acid. The whole extraction protocol was performed in the class 1000 clean room of the MeGic laboratory (Metallomics and Geochemistry Research lab at the Department of Chemical and Geological Sciences, UNIMORE), under a laminar flow hood. Extracted peptides were dried and resuspended in a mixture of water:acetonitrile:formic acid 95:3:2 and analyzed using Nano UHPLC Ultimate 3000 coupled to an Exploris 480 Hybrid Quadrupole-Orbitrap Mass Spectrometer [see (*87*)] at the CIGS of UNIMORE. Bioinformatic data analysis of raw datasets (.RAW format) was performed using PEAKS XPro (PEAKS Studio 10.6 build 20201221) with default settings and using Trypsin set in the unspecific digestion mode. Tolerances were set at 10 parts per million and 0.02 Da for parent and fragment mass error. Deamidation (NQ), hydroxylation (ALL), and oxidation (M) were set as variable PTMs with a maximum of 3 PTMs per peptide. Peptide length was set above 7 amino acids. Peptides were searched against an Asian Elephant NCBI reference proteome (EleMax_GCF_024166365, downloaded 31 October 2024) modified with FASTA formatted UNIPROT sequences of AMELX, AMELY, MMP20, AMBN, and ENAM gene products from *L. africana*, *Elephas maximus*, *Bos taurus*, *Sus scrofa*, and *Homo sapiens.* A decoy database was used and data were filtered at 1% false discovery rate; other settings were left as default. AMELY and AMELX sequences for *E. maximus* were obtained translating from genome sequences. AMELX and AMELY quantitation was obtained by pooling all extracted ion current measurements from peptides unique to each respective gene product (*85*). See fig. S8 for validation of sex estimation.

### Sr isotope analysis by (LA-)MC-ICPMS

After cutting the molars in half, the two longest and macroscopically best-preserved enamel lamellae per tooth (fig. S7) were selected from 10- to 15-mm-thick tooth slabs for isotope analysis to minimize diagenetic alteration effects and obtain the longest lifetime representation. Unfortunately, there is no information available about *P. antiquus* tooth formation time. However, based on what is known from modern elephants and other extinct proboscidean species, each lamella of a proboscidean molar records most of the ontogenetic time period, with variations depending on molar type (*21*, *23*, *72*, *83*, *88*). In situ Sr isotope analysis was carried out using a Thermo Fisher Scientific NeptunePlus MC-ICPMS coupled to a RESOlution LA system (ArF, 193 nm) with a Laurin Technic S155 two-volume laser-ablation cell, all at the Frankfurt Isotope and Element Research Center (FIERCE, Goethe University Frankfurt), and overall follows the methodology described in Müller and Anczkiewicz (*89*). Measurements were conducted in the innermost enamel along the enamel-dentine junction [see (*50*); fig. S3]. One short line (approximately 5 mm) per tooth was additionally measured in the dentine to monitor the potential diagenetic effects, as dentine is more prone to diagenetic alteration (*90*). Sample pre-ablation was set at 9 mm/min, using 130-μm laser spot size and a repetition rate of 20 Hz. The ablation scan speed ranged between 1 and 1.5 mm/min, using 108-μm spot size and a repetition rate of 15 Hz. The integration time was 4 s. All LA-MC-ICPMS analyses were performed in robust plasma mode, i.e., ThO/Th ~ 0.5% and Th/U > 95%. Two isotopically homogeneous marine in-house reference materials were used, with modern seawater ^87^Sr/^86^Sr values (~0.7092). A shark tooth enameloid yielded an average ^87^Sr/^86^Sr of 0.70925 ± 0.00004 (2SD, *n* = 16) and ^84^Sr/^86^Sr of 0.0565 ± 0.0001, while a marine *Tridacna* sp. bivalve shell sample yielded an average ^87^Sr/^86^Sr of 0.70920 ± 0.00010 (2SD, *n* = 4) and ^84^Sr/^86^Sr of 0.0565 ± 0.0001.

For solution-based Sr isotope analysis, tooth enamel from different mammalian families found in both NN-1 and NN-2 sites (i.e., *Bovidae*, *Canidae*, *Cervidae*, *Elephantidae*, *Equidae*, *Felidae*, *Hyaenidae*, *Rhinocerotidae*, and *Ursidae*) was sampled to build a Sr isotope baseline for the local bioavailable ^87^Sr/^86^Sr. Whenever possible, approximately 10 mg of enamel powder per tooth was drilled from the whole crown surface to obtain average ^87^Sr/^86^Sr values representing the entire formation period and reduce potential seasonal bias in possibly migratory species. This was not the case for the “bulk” elephant samples, for which enamel powder was collected close to the occlusal surface due to the size of the tooth samples. Aliquots of all baseline powder samples were dissolved in concentrated HNO_3_ (approximately 1 mg), and strontium separation from the matrix was performed using an automatized prepFAST MC (ESI Elemental Scientific) system following the elution protocols described in Romaniello *et al*. (*91*) and Weber *et al*. (*92*). Procedural blanks and aliquots of NIST SRM1486 cow bone meal and NIST SRM1400 cow bone ash reference materials were processed together with the samples. The pure Sr fractions were diluted to reach a concentration of 30 ng/ml and analyzed on a Thermo Neptune plus MC-ICP-MS coupled to an ESI Apex Omega HF desolvating system following (*92*). Mass bias normalization was performed through the exponential law, using an ^88^Sr/^86^Sr ratio of 8.375209. Samples were bracketed by and normalized to a NIST SRM 987 ^87^Sr/^86^Sr value of 0.710248 (*93*). Procedural Sr blanks yielded an average mass of 0.10 ± 0.06 ng (*n* = 4), negligible when compared to the minimum Sr sample size of 32 ng (average sample size, 110 ng). SRM1400 cow bone ash and SRM1486 cow bone meal yielded average values of 0.713108 ± 31 (2SD, *n* = 2) and 0.709293 ± 21 (*n* = 5), respectively. These values are in good agreement with previously reported ratios for SRM1400 of 0.713190 ± 220 and for SRM1486 of 0.709340 ± 270 (*94*).

### Oxygen and carbon isotope analyses of enamel bioapatite

One elephant molar with more radiogenic ^87^Sr/^86^Sr ~ 0.713 (832A) and one with less radiogenic ^87^Sr/^86^Sr ~ 0.710 (E22) were selected for δ^13^C and δ^18^O analysis of the structurally bound carbonate moiety in the enamel bioapatite. The enamel of one lamella per tooth was sequentially sampled using a microdrill (Merchantek). For specimens 832A and E22, samples ~1.5 mm wide were drilled at ~1.5 mm distances along the enamel-dentine-junction following the laser line, for a total of 63 and 95 samples, respectively. The stable isotope analyses were carried out at the inorganic stable isotope laboratory of the Department of Climate Geochemistry at the Max Planck Institute for Chemistry in Mainz. Samples were analyzed on a Thermo Delta V mass spectrometer, interfaced with a Gasbench-II + coldtrap cryofocusing system, following the analytical procedure of Vonhof *et al.* (*95*). About 50 to 100 mg of powdered tooth enamel sample, placed in a He-filled 12-ml exetainer vial with a septum cap, was digested in water-free H_3_PO_4_ at a temperature of 70°C. Subsequently the CO_2_-He gas mixture was transported to the GASBENCH in 5.0-grade helium carrier gas. In the GASBENCH, water vapor and various gaseous compounds were separated from the He-CO_2_ mixture before sending it to the mass spectrometer for isotope analysis. Isotope values are reported as δ^13^C and δ^18^O values relative to VPDB. A total of 20 replicates of two in-house tooth enamel standards were analyzed in each run of 35 samples. The AG-Lox tooth enamel standard has a δ^13^C value of −11.58‰ and a δ^18^O value of −1.42‰, and the tooth enamel standard MAMMY has a δ^13^C value of −13.49‰ and a δ^18^O value of −14.76‰, all on the VPDB scale. Standard material weights are chosen so that they span the entire range of sample weights in the run. The 11 replicates of AG-Lox in each run are used for the correction of isotope effects related to sample size, and for scale conversion. After size effect correction, the reproducibility of AG-Lox was 0.10‰ (1SD) for δ^13^C and 0.16‰ (1SD) for δ^18^O. MAMMY is used as a control standard, and yielded a δ^13^C of −13.54‰ (±0.11‰, 1SD, *n* = 45) of and a δ^18^O of −14.97‰ (±0.24‰, 1SD, *n* = 45), fully consistent with the accepted values.

### Sr-O-C data comparison

Given the lack of species-specific information about tooth formation time of *P. antiquus*, we used the large-bodied *Mammuthus columbi* as the reference species. Therefore, the expected maximum formation time of a molar tooth was 10 to 11 years (*23*, *27*, *72*), which is about one-seventh of the estimated lifespan for *P. antiquus* (*15*). All the teeth analyzed but E22 were worn. Hence, we decided to calculate a modeled age for E22 only, which was completely formed but still not fully erupted and thus unworn. For this individual, we estimated the tooth formation time using the model of Dirks *et al*. (*23*), based on the distance from the cusp.

To be able to align and statistically compare (i.e., linear regression) on a common temporal scale the isotopic data obtained at different spatial resolutions (higher for Sr and lower for O and C), these intra-tooth profiles need to be at the same resolution. To achieve this, the Sr data measured at a higher spatial resolution were resampled at a lower resolution, similar to that of the O and C isotope profiles (i.e., averaging the ^87^Sr/^86^Sr signal bins every ~1.6 mm). Then, a LOESS smooth function (span = 0.05) was fitted to the resampled Sr isotope data, and Sr values were interpolated at the same cusp distances as O and C isotope samples (fig. S9). Last, Sr versus O and Sr versus C isotope data were calculated through linear regression models, to search for eventual relationships between the different isotope systematics. In addition, a cross-correlation analysis was performed to evaluate the signals’ lag of the time series, using the ccf() function in R (fig. S10).

### Geostatistical framework

A Sr isoscape of NE Germany has been developed to test the provenance/mobility of the elephants. We used a random forest algorithm (randomForest package) with ecogeological external predictors (*n* = 23) as described in Bataille *et al*. (*96*) to build a predictive model of the Sr isotope distribution for the Neumark-Nord area. Reference Sr isotope values (*n* = 258) were collected mainly from Käßner *et al*. (*30*) and from other studies (table S5). A median value of 0.7104 was used for Neumark-Nord, calculated from bulk enamel Sr isotope data of the contemporaneous fossil mammal fauna (*n* = 116). We acknowledge that the species used to construct the local Sr baseline may have had relatively large home ranges. However, the dataset appears consistent both internally (table S3) and with the expected local geological signature (Fig. 3). Moreover, the use of the median is a standard approach to derive a robust central value while minimizing the influence of potential outliers (*97*). The final Sr isotope map has been cropped at approximately 300 km around the archaeological site (longitude: 8–16°E and latitude: 49–54°N) and the modeled ^87^Sr/^86^Sr ranged between 0.7074 (min) and 0.7199 (max). A 10-fold cross-validation was used to estimate the power of the prediction, evaluated as RMSE (root mean square error) and *R*^2^ (explained variance). These resulted as RMSE = 0.0015 and *R*^2^ = 0.75. To build a spatial-uncertainty map, we used a quantile random forest regression (ranger package), then halving the *q*_0.84_ − *q*_0.16_ difference (i.e., lower and upper limits of a ∼68% interval). The prediction error map ranged between 0.0002 and 0.0062 (fig. S11) (*48*).

The ^87^Sr/^86^Sr ratios extracted from the LA profiles were compared with the isoscape map using a Bayesian probabilistic approach and the assignR package in R (*98*). We ran this method on specific values of each profile as follows: (i) minimum and maximum values; (ii) modes calculated (i.e., the “most common” isotope values observed along the profile) from the density function [density in *R*, bandwidth picked automatically with the method of Sheather and Jones (*99*)] as local maxima. Then, for each tooth, the provenance for all these values (min, max, and modes) was evaluated with assignR (*98*). The prior probability of this Bayesian approach assumes that all grid cells are equally likely locations of origin of these samples. The posterior probability of origin is computed at each grid cell, returning a raster object, which contains one probability density surface per sample with its likely provenance. Top 5% probability values were calculated and clustered to obtain overview-maps of residence areas for each individual. Using the function st_intersection (sf R package), the relative overlap of these areas was calculated for pairwise elephant molar comparisons.

## Supplementary Materials

### The PDF file includes:

Supplementary Text Figs. S1 to S11 Table S1 Legends for tables S2 to S5 References
