# Supplementary material for: Life histories of straight-tusked elephants from the Last Interglacial Neanderthal site of Neumark-Nord (~125 ka)
Source: Sci Adv. 2026 Mar 13;12(11):eadz0114. doi: 10.1126/sciadv.adz0114 (PMC12985690; doi:10.1126/sciadv.adz0114)
Supplement: Supplementary file 1 — Supplementary Text Figs. S1 to S11 Table S1 Legends for tables S2 to S5 References [file sciadv.adz0114_sm.pdf]

Science Advances
AAAS

# Supplementary Materials for

## Life histories of straight-tusked elephants from the Last Interglacial Neanderthal site of Neumark-Nord (~125 ka)

Elena Armaroli *et al.*

Corresponding author: Elena Armaroli, elena.armaroli@unimore.it; Federico Lugli, federico.lugli@unimore.it

*Sci. Adv.* **12**, eadz0114 (2026)
DOI: 10.1126/sciadv.adz0114

**The PDF file includes:**

Supplementary Text
Figs. S1 to S11
Table S1
Legends for tables S2 to S5
References

**Other Supplementary Material for this manuscript includes the following:**

Tables S2 to S5

## Supplementary Text

### The Last Interglacial lake landscape of Neumark-Nord – Recovery – Investigation – Results – Provenance of Material

The Last Interglacial (Eemian) basins Neumark-Nord 1 and 2 (NN-1, NN-2), filled with fossiliferous limnic sediments, are located in the former open-cast lignite mine of Mücheln in Saxony-Anhalt, Germany. NN-1 was discovered in 1985 and was investigated during active mining until the mid-1990s by a team led by Jena archaeologist Dietrich Mania. With the end of mining activities and the beginning of reclamation works, Mania also discovered basin NN-2 in 1996. From 2004 to 2009, the Eemian infill of basin NN-2 was excavated in year-round campaigns by a team led by the present authors LK, SGW, and WR, in close collaboration with the State Heritage Office of Saxony-Anhalt [Landesamt für Denkmalpflege und Archäologie, Sachsen-Anhalt (LDA-LSA)] in Halle, Germany. The archaeological excavations included a field school, which trained over 175 international students, and were accompanied by a comprehensive multidisciplinary study of the basin infill. Today, both basins are submerged and lie below the water table in a constructed artificial lake (Geiseltalsee) in the former lignite mine.

With a size of approximately 30 ha the Neumark-Nord exposure provides a paramount insight into the Last Interglacial ecosystem in central Germany. A first thorough documentation of Neumark-Nord 1 was presented by Mania *et al.* in 1990 (101) followed by 2 decades of publications on archaeological, paleontological, geological and palaeoecological investigations and analyses at Neumark-Nord. A comprehensive overview of the NN-1 record is provided by Meller (102) and Mania (103). Results of multidisciplinary studies of the infill of basin NN-2, including a range of dating studies, can be found in Sier *et al.* (104), Pop and Bakels (52), Strahl *et al.* (105) and Gaudzinski-Windheuser and Roebroeks (6), with the two latter publications containing a first stratigraphic correlation of the NN-1 and NN-2 basin infills. A more nuanced synchronization of both basins including temporal and spatial sequencing of find deposits

based on palaeoecological, sedimentological, geomorphological and hydrological evidence is established in Kindler *et al.* (9).

In short: Sandwiched between the Saalian till in the underground and Weichselian loess on top, both basins contain a sequence of lake sediments containing a pollen record of the full Eemian forest succession developing on late Saalian deposits and covered by the onset of the Early Weichselian, without major hiatus. The temporal resolution of find deposits is given by the duration of respective Pollen Assemblage Zones (PAZ) (106, 107), which can be narrowed down by sedimentation rates of different layers, water table fluctuations within the PAZ, micromorphology and additional botanical evidence such as tree ring counting. New estimates on the duration of the Eemian (108) do not conflict with the temporal resolution of the material presented in this study. Important recent discoveries include the anthropogenic fire regime during the first part of the Eemian (6) , Neanderthal involvement in the formation of the fallow deer thanatocoenosis mostly during PAZ V at NN-1 (12, 109), massive exploitation of elephants, with most individuals deposited during a short regression phase during PAZ VIa at NN-1 (lower shore horizon) (12, 18), and evidence for intense bone grease rendering (NN2/2B – fat factory) at the margin of NN-2 during a transgression phase of PAZ IVa (28).

The elephant specimens sampled in this study were retrieved from sediments of the first part of the Eemian (PAZ I-IVa), deposits underlying the lower shore horizon, the lower shore horizon at NN-1 and from the “fat factory” at NN-2. Based on PAZ duration these finds cover a maximum time period of 1950 years. Including sedimentation rates estimations for the different layers from PAZI–IVa the specimens analyzed in this study were recovered from deposits which represent about 1050 years of time. More details on the spatio-temporal provenance of the sampled elephants are provided in the following section.

All information, documentation, and materials from Neumark-Nord are the property of the State of Saxony-Anhalt, with the LDA-LSA serving as the responsible administrative authority. The material remains part of several ongoing research projects led and organized by SGW, WR, LK and the LDA-LSA.

## Spatio-temporal provenance of the elephant teeth analyzed

The four specimens of *P. antiquus* analyzed in this study were retrieved from early Eemian deposits in the Neumark lake landscape, spanning approximately 2000 years (Figs. S1 and S2). The molar from elephant complex (E)5 belongs to the protocratic phase (PAZ I-III of the Eemian) and was found in the upper part of the fine warved gyttia layer (NN1.4.), representing a first Eemian lake phase at Neumark. From E5, only the molars could be recovered and no bones were retrieved due to mining activities. However, another carcass (E6), less damaged by bucket wheel excavators, was excavated in this deposit and shows anthropogenic modifications on the bone surfaces.

Regression of the water table opened an up to 300 m wide sandy margin in basin NN-1 (lower shore horizon NN1.6.1) with the beginning of the mesocratic phase (PAZIVa). Here, 24 elephant complexes (E) were originally described (102), representing minimally 39 individuals, with most of the carcasses displaying cut marks. Elephant complex E22 represents 4 individuals (A-D), one 26-year-old male and three individuals older than 26 years. The bones of these three individuals inhibit a clear sex determination. The duration of this regression phase is calculated to approximately 300 years.

At NN-1, an additional 10 individuals were deposited in the basin silts during PAZ IVa. As these individuals also show signs of anthropogenic modification, it is likely that the carcasses became submerged quickly after deposition on dry land, and are best attributed to the subsequent transgression phase, which led to the complete submergence of basin NN-1. During this transgression phase, the main find layer NN2/2B at the margin of basin NN-2 developed over a maximum duration of about 288 or 455 years, based on sedimentation rate calculations and PAZ attribution of the sedimentary sequence. More than 20000 lithic artefacts and 120000 bone specimens, spread over an area of less than 500 m², indicate a high and prolonged activity zone of Neanderthals. The minimal number of individuals (MNI) is 172, including 56 horses, 54 cervids, 45 aurochs, and 2 straight-tusked elephants. While the elephant complexes in basin NN-1 represent an individual, autochthonous thanatocoenosis, NN2/2B must be considered an allochthonous thanatocoenosis, a site where carcasses or parts of them were repeatedly transported. The molars NN2/2/2719 and NN2/2/3832 are part of this extensive find accumulation.

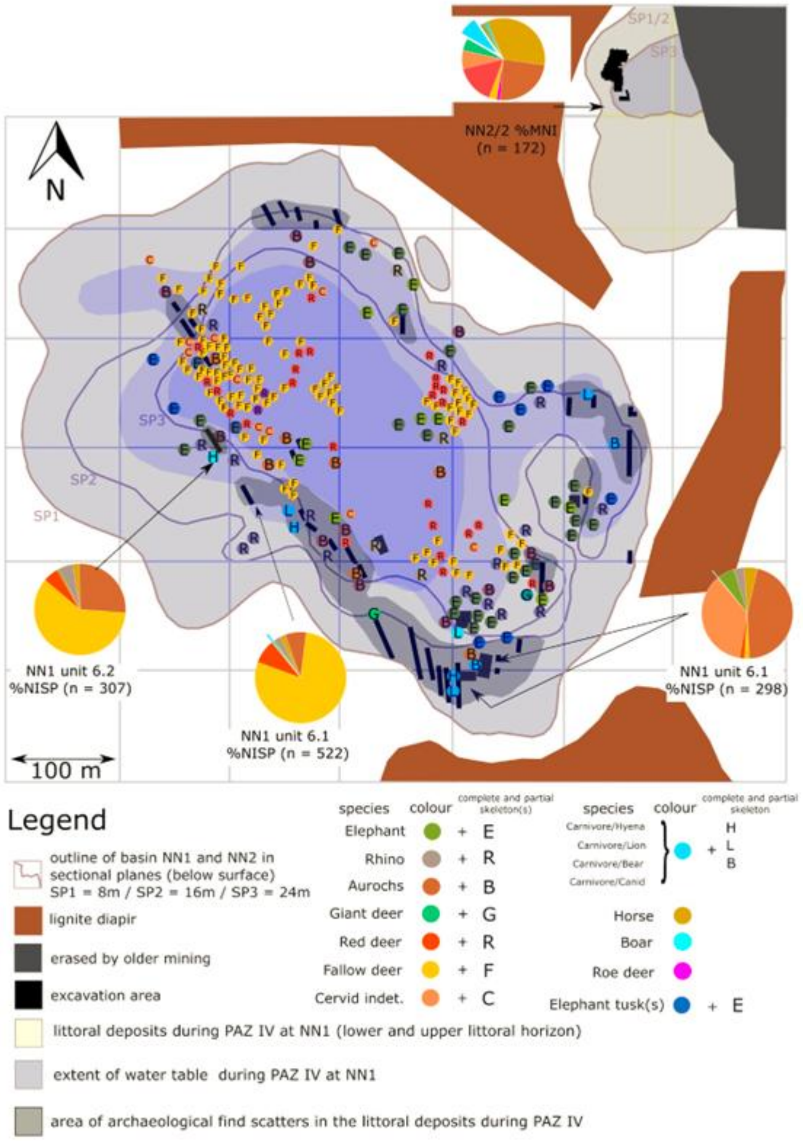


**Fig S1**. Outline and extent of basin NN-1 and NN-2 in the former lignite mine Mücheln, including a synopsis of all (partial) skeleton remains within basin NN-1, and proportions of mammal species from individual excavation areas at NN-1 and NN-2 (updated version from (9)).

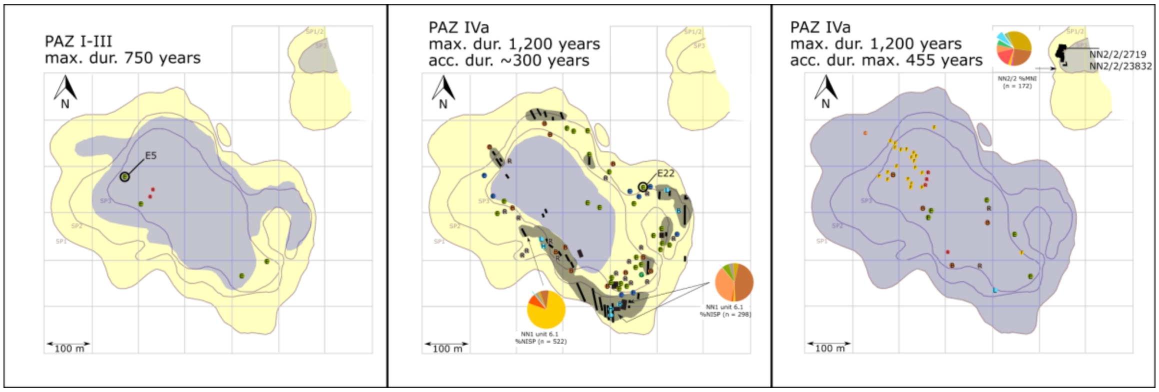


**Fig S2.** Provenance of the sampled elephant teeth. Indicated are temporal associations in terms of maximal duration of PAZ (Pollen Assemblage Zones, see (106-108), maximal duration (max. dur.) of bone accumulation within the find layers, water tables within the basin, and associated (archaeological) finds.

## Age at death of the sampled elephants: timing of the dental isotopic signals based on ontogenetic considerations

Proboscideans develop six molars which are replaced consecutively during lifetime. Phylogenetically, the first three molars (M1-M3) are deciduous, while the last three are permanent (M4-M6). Each tooth is built up of isolated lamellae which are welded together during tooth formation. Generally, the number of lamellae increases with each consecutive tooth. A comprehensive summary of elephant tooth morphology, development, wear and replacement as well as a guide to distinguish individual teeth is provided by (110). Based on a collection of lower jaws from African elephants (*Loxodonta africana*) Laws (83) established 30 groups of successive tooth development, eruption, wear stages, and replacement which allow estimation of age at death based on individual teeth. His scheme is widely adopted for aging recent and fossil specimens, with individual age estimates continually updated since then (e.g., 110-113).

Using the 'Laws criteria' to group tooth wear and the revised age estimates provided by Lee *et al.* (112) elephant E5 died at 16 years, E22 at 24 years, NN2/2/2719 at 47 years, and NN2/2/3832 at 30 years. Since the isotope signal is recorded during stages between tooth formation, mineralization, root closure, and initial wear of the sampled lamellae, an earlier

stage in the individuals' life history is recorded. Thus, the isotope sample from individual E5 reflects the immature stage, from E22 the adolescent stage, from NN2/2/2719 the young to midlife adulthood, and from NN2/2/3832 the adolescent to young adulthood stage (Table S1).

| Sample ID | Site | US | This work ID | Tooth type | Age Group (83) | Age at death (112) | Tooth formation (112) | Tooth lamellae in wear (112) | Life history stage at death (114) | Life history stage at isotope imprint (114) |
|---|---|---|---|---|---|---|---|---|---|---|
| E5 | NN-1 | NN1.4 lower gyttja | elef11B | Upper M4 | XII | 16 y | 4 y | 14 y | Adolescent | Immature |
| E22 | NN-1 | NN1.6.1 lower littoral | elefE22 | Upper M5 | XVI | 24 y | 14 y | 21,5 y | Young adult | Adolescent |
| NN2/2/2719 | NN-2 | NN2/2B | elef2719A | Lower M6 | XXVI | 47 y | 27 y | 41 y | Senior adult | Young – midlife adult |
| NN2/2/3832 | NN-2 | NN2/2B | elef832A | Upper M5 | XIX | 30 y | 14 y | 27 y | Young adult | Adolescent – young adult |

**Table S1. Aging data obtained for the four individuals.**

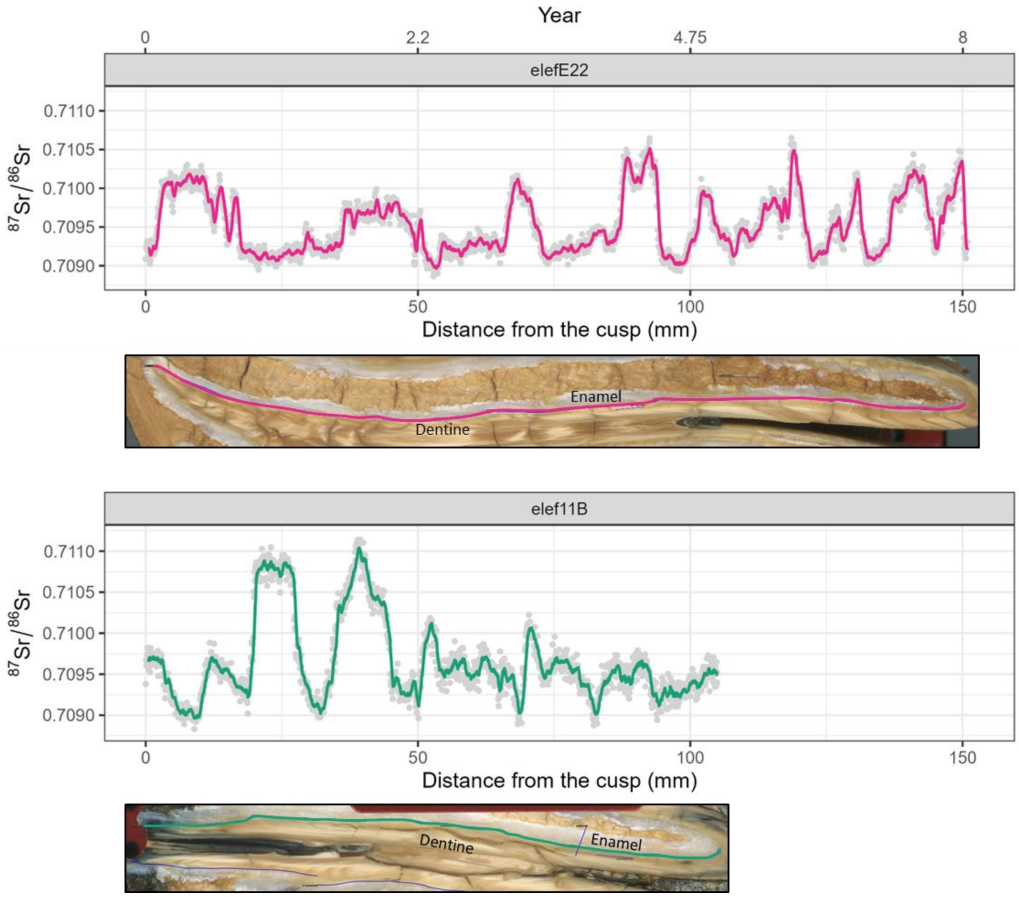


**Fig S3. $^{87}Sr/^{86}Sr$ profiles of *P. antiquus* tooth enamel (samples elefE22 and elef11B) obtained through *in situ* LA-MC-ICPMS.** The Sr isotope composition was measured *in situ* continuously along the enamel-dentine junction of the whole lamellae length. Note the corresponding years of life are shown for the individual E22, the only one unworn because not fully erupted. The modelled age was calculated following (23).

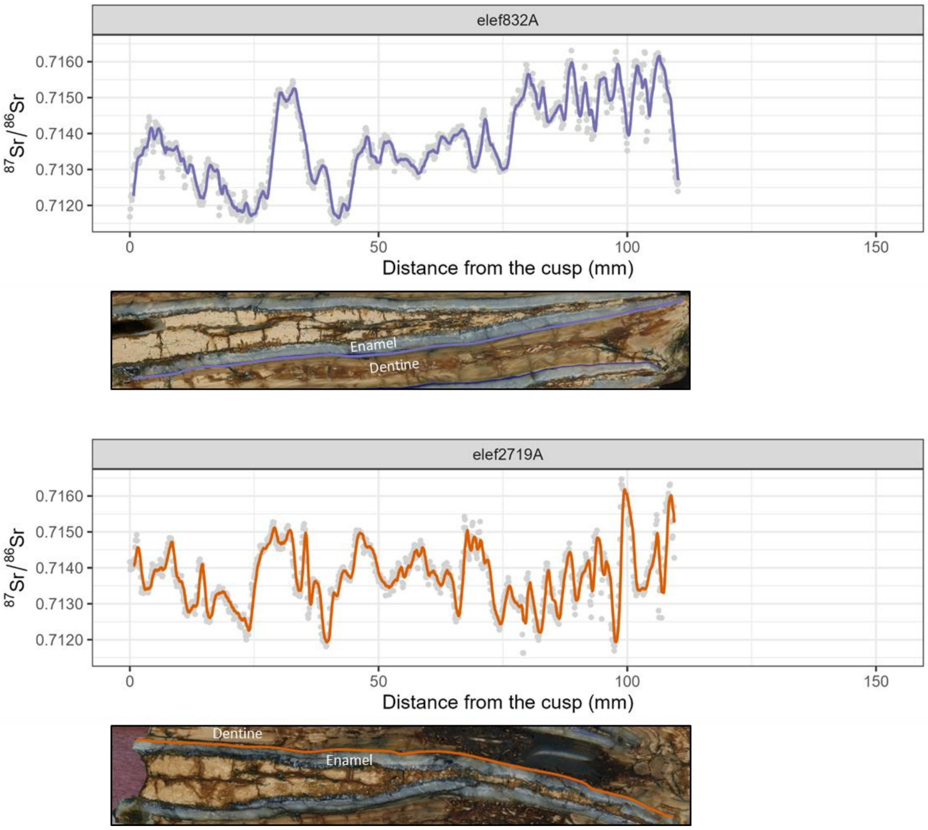


**Fig S3 (*continue*). $^{87}Sr/^{86}Sr$ profiles of *P. antiquus* tooth enamel (samples elefE22 and elef11B) obtained through *in situ* LA-MC-ICPMS.** The Sr isotope composition was measured *in situ* continuously along the enamel-dentine junction of the whole lamellae length.

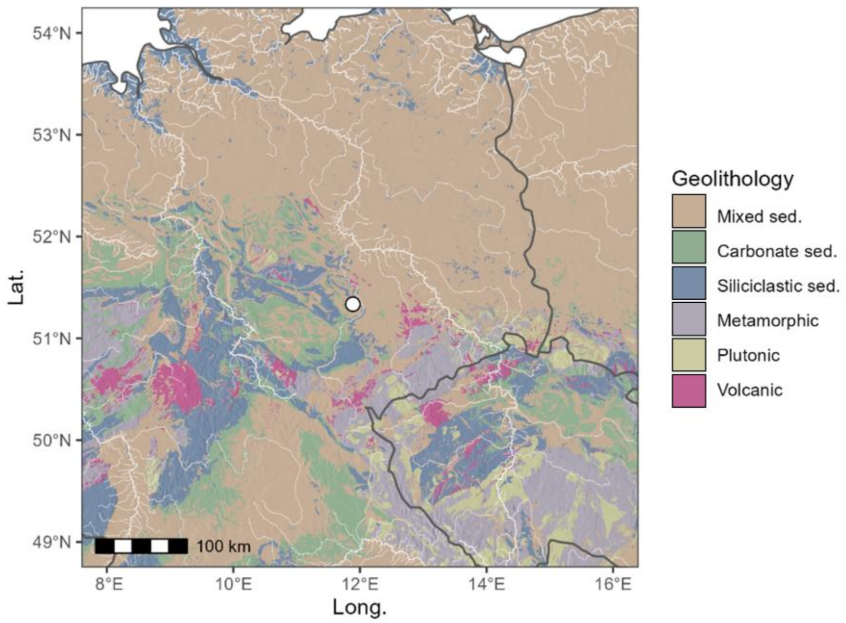


**Fig S4. Simplified geological map**. Geolithological data are from GliM (115); for a geographical reference see Fig. 1 of the main text. Rivers (white) are from (116). The white circle marks the site of Neumark-Nord. The map was made with R (version 4.0.5).

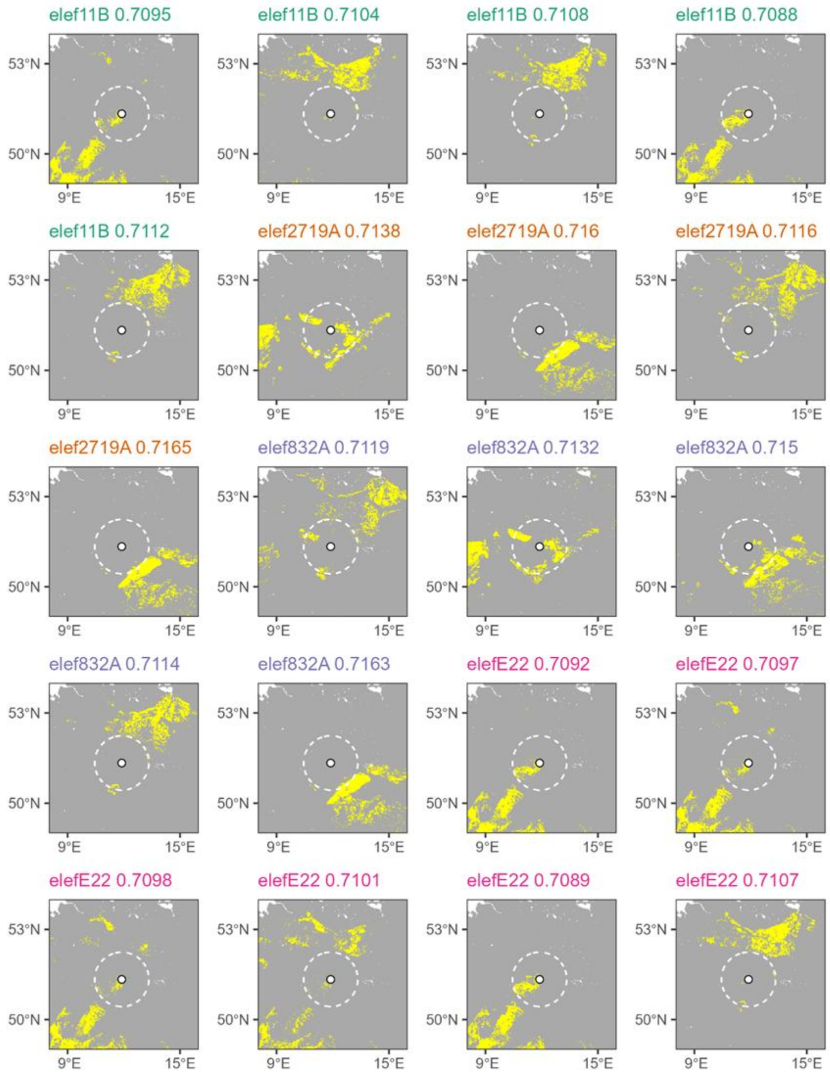


**Fig S5. Elephants' provenance probabilities estimated through Bayesian approach.** The white dot marks the site of Neumark-Nord. Yellow areas are top 5 % probability estimates. The white dashed circle marks 100 km of radius around the site. Maximum, minimum and modal $^{87}Sr/^{86}Sr$ values were tested for each sample (see Fig. 5 of the main text). The geographical framework is reported in Fig. 3 of the main text.

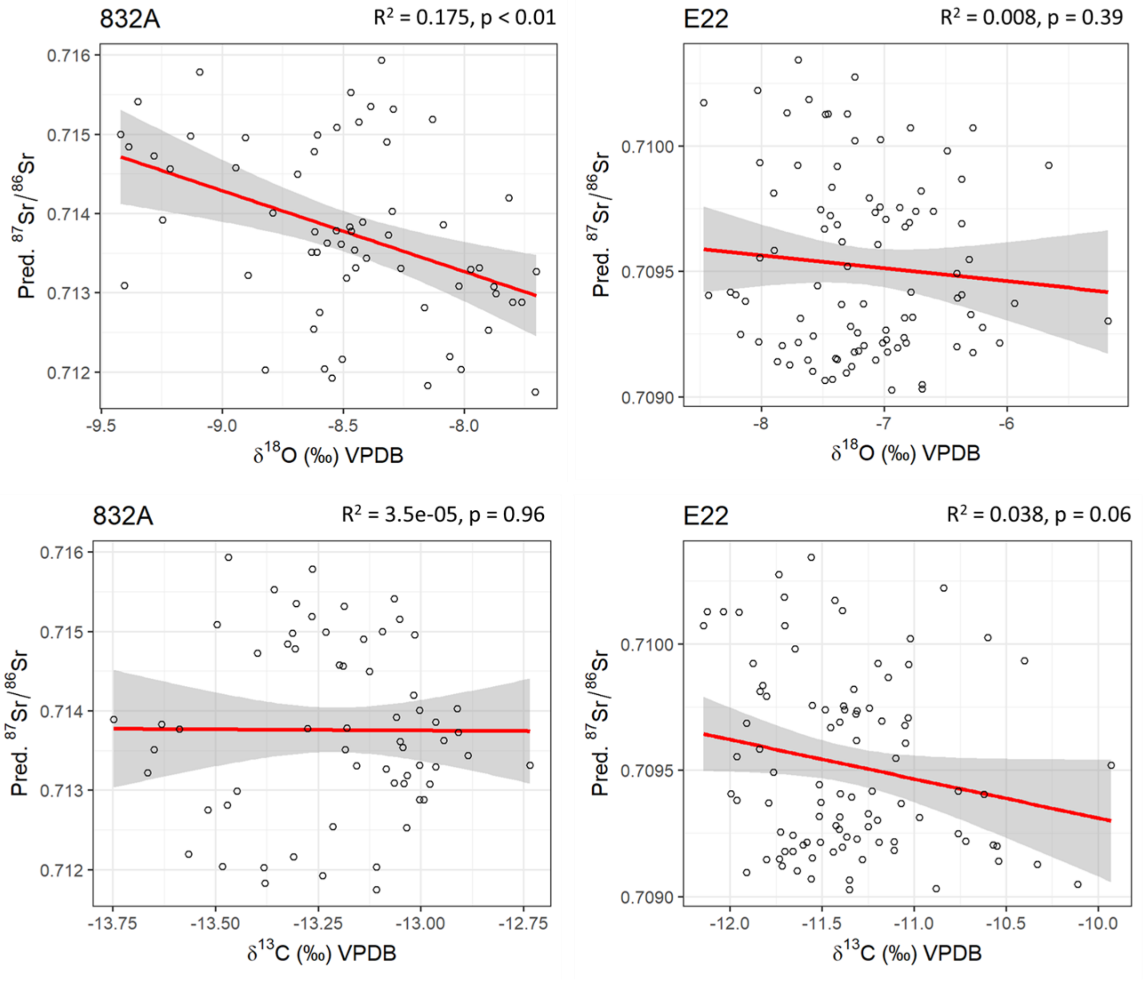


**Fig S6. Comparison of Sr and O and C isotope data.** Resampled Sr isotopes were compared with O-C isotope data. Red lines are linear models fitted through the data; the grey ribbon is the standard error of the model.

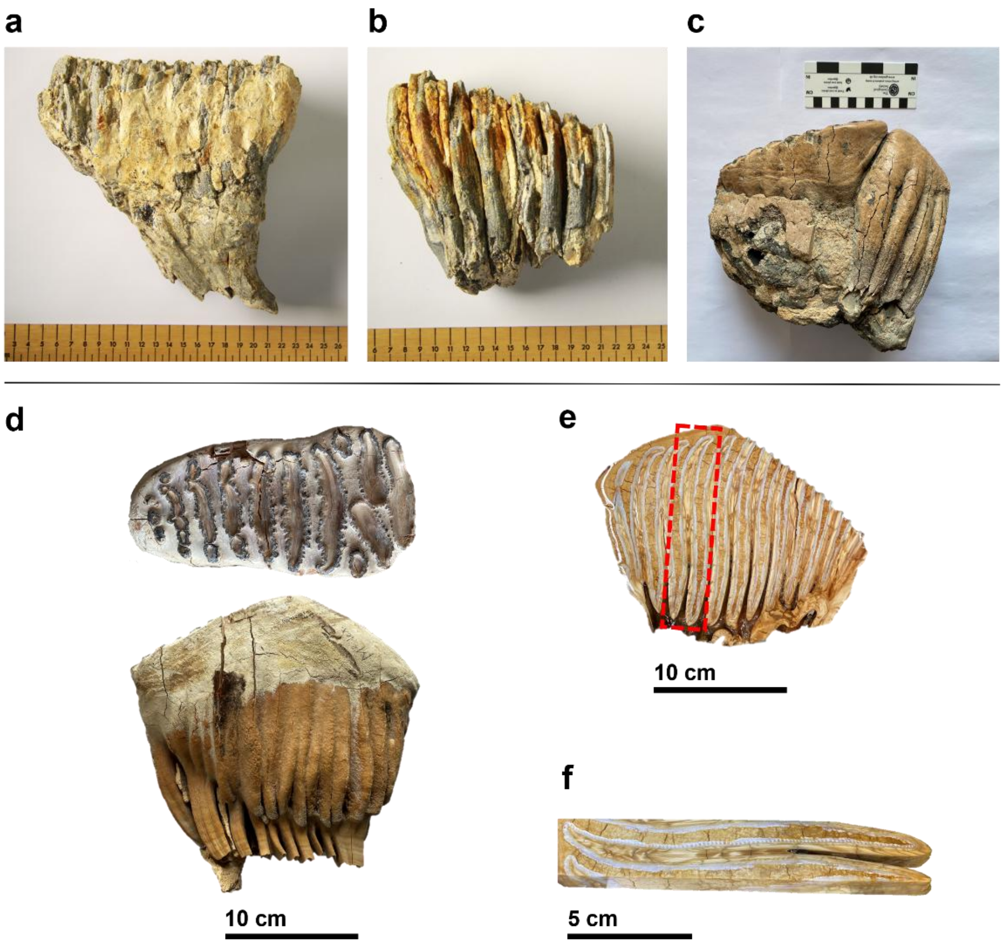


**Fig S7. Straight-tusked elephant molars analyzed in this study and cutting workflow: a)** elef2719A; **b)** elef832A; **c)** elef11B; **d)** elefE22 before cutting; top: occlusal surface, bottom: left side view with roots at the bottom; **e)** elefE22 cut in half, occlusal surface top-right. The dashed red line indicates the lamella selected for isotope analysis; **f)** Cut lamella after sampling for isotope analysis.

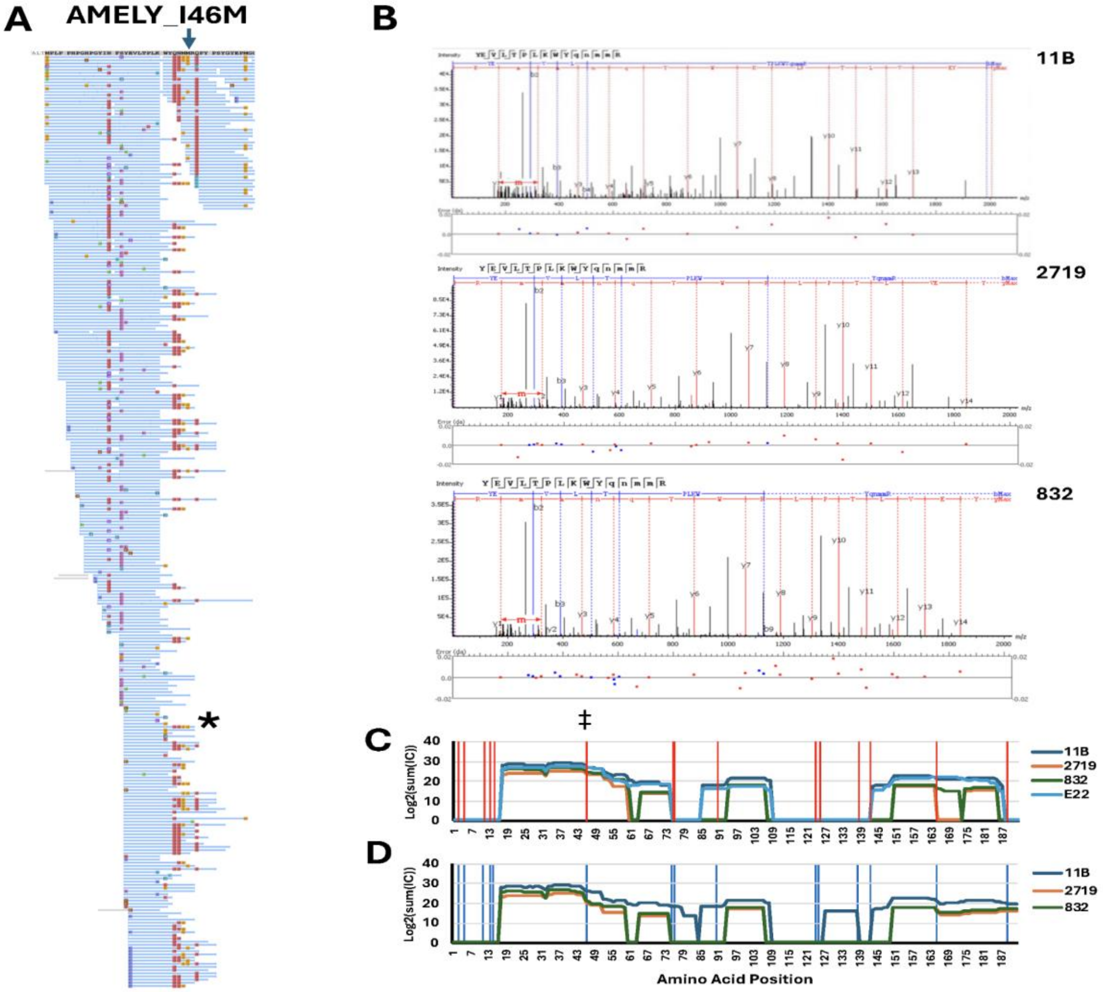


**Fig S8. Validation of male sex estimates.** Enamel from each *P. antiquus* individual was processed proteomically, submitted to an Exploris 480 LC/MS/MS, and resulting fragmentation mass spectra matched to *E. maximus* amelogenin (AMELX and AMELY) gene products using PEAKS Xpro and quantified. **A)** A subset of peptide spectra matches, indicated by light blue bars, corresponding to the distinctive 'MM' motif in the AMELY gene product at position 45 and 46, from individual 11B is illustrated. **B)** Mass Spectra from an unambiguous spectrum from the AMELY peptide (*, YEVLTPLKWYQ(d)N(d)M(ox)M(ox)R), for three male *P. antiquus* individuals (11B, 2719, 832). Note that all three spectra contain double mercaptoethanol (64 Da) immonium ion losses. The signal depth of coverage (log2(sum(IC)) of each amino acid for AMELX **(C)** and AMELY **(D)** are plotted for all individuals, with single amino acid variants specific for each gene product indicated (AMELX = red, AMELY = blue, methionine 46 = ‡).

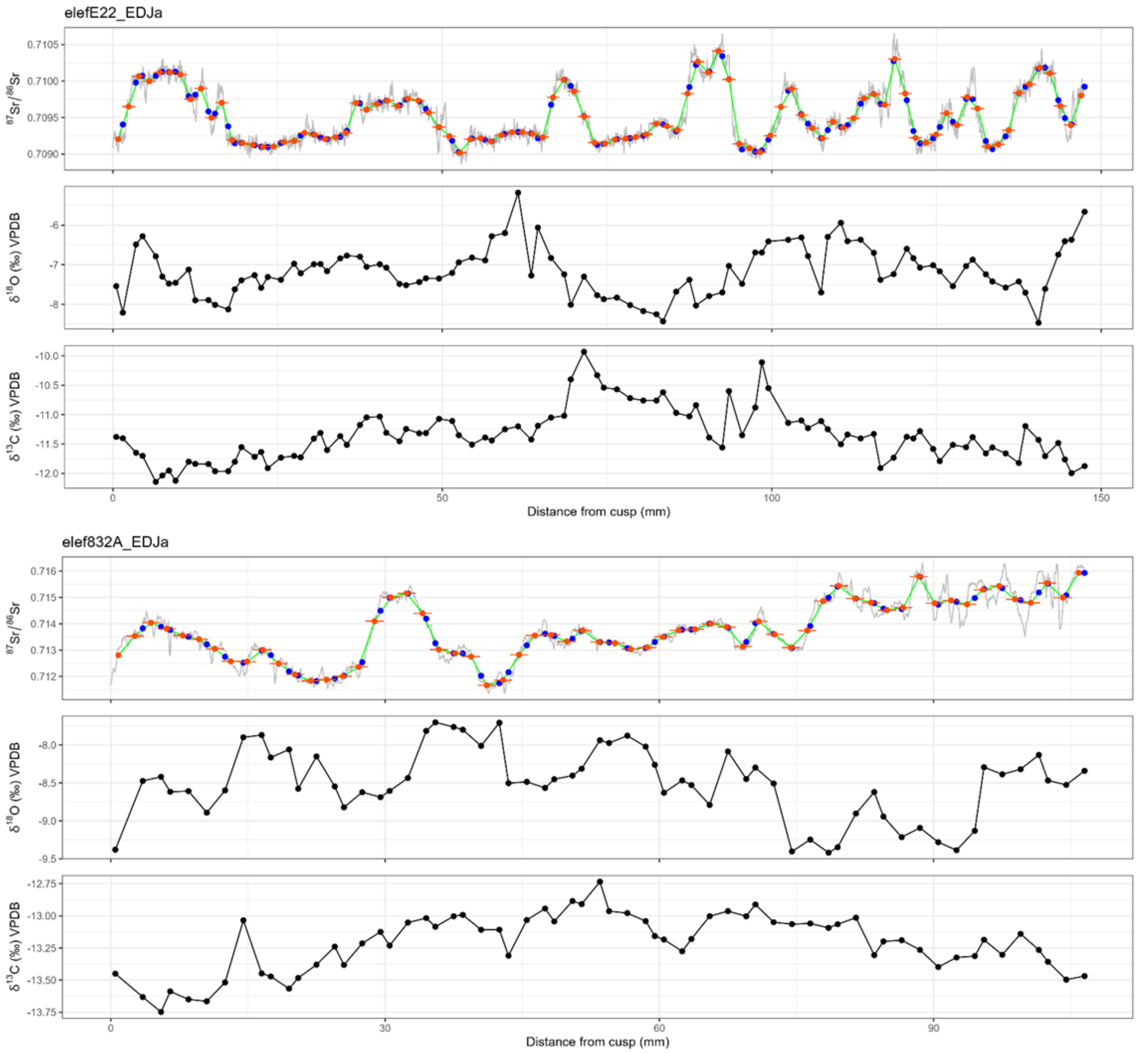


**Fig S9. Sr isotope profiles resampling.** To obtain Sr isotope data at the same resolution of the C and O data, the signals are averaged every ~1.6 mm. A LOESS smooth function (span = 0.05) is fitted to the binned Sr isotope data and Sr values are interpolated at the same cusp distances as C and O isotope data. In the $^{87}Sr/^{86}Sr$ graphs, red circles are the binned data, green profiles are the smooth functions, blue circles are the resampled data at C and O cusp distances.

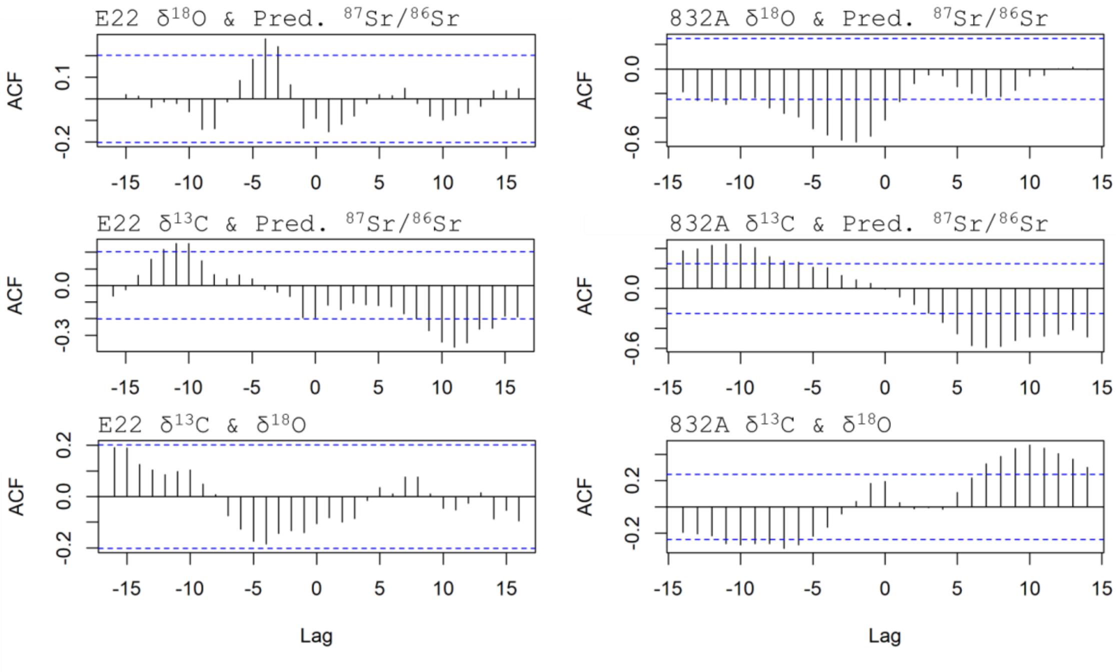


**Fig S10.** Cross-correlation analysis of Sr-C-O time series for samples E22 and 832A. Blue dashed lines represent 95% confidence intervals. ACF = autocorrelation function. For individual E22, most ACF values are within the confidence intervals and $-0.5 < r < 0.5$, thus considered not significant. ACFs of individual 832A reveals a negative (auto)correlation ($r < -0.5$) of $\delta^{18}O$ and $^{87}Sr/^{86}Sr$ at a lag of -2, indicating that strontium variations anticipate oxygen by approximately 2–4 months. Additionally, $\delta^{13}C$ and $^{87}Sr/^{86}S$ show a similar negative correlation at a lag of 7, suggesting that carbon changes precede strontium by about 6–9 months. Yet, considering the limited intra-tooth variation of $\delta^{13}C$ for 832A (< 1‰) and the relatively long lag in the context of an individual's lifespan, we caution against drawing strong inferences regarding the carbon–strontium relationship.

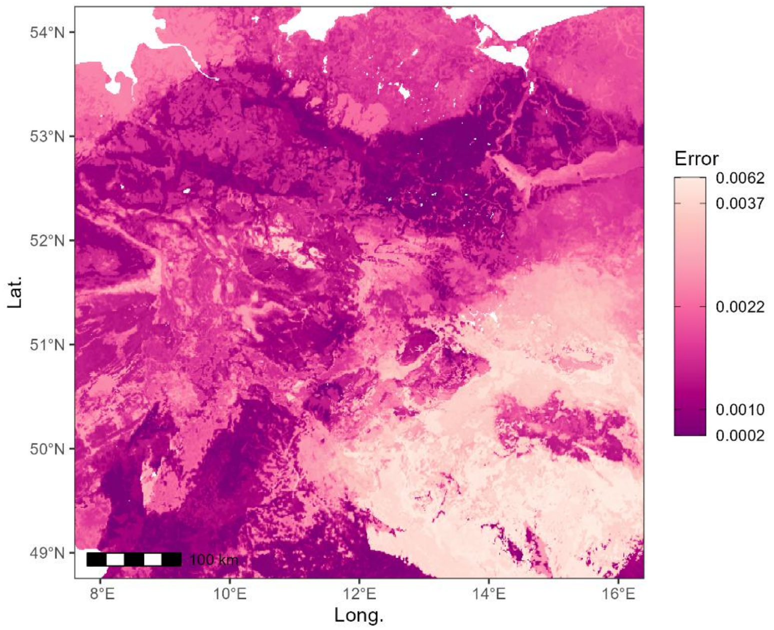


**Fig S11. RF isoscape spatial-uncertainty map**. The map was built through a quantile random forest regression (*ranger* package) to estimate the prediction power of the model. The prediction error ranges between 0.0002 (purple) and 0.0062 (light pink).

**Supplementary Tables**

**Table S2.** Sr isotope LA-profile data.

**Table S3.** Sr baseline samples.

**Table S4.** Summary of isotopic and proteomic results.

**Table S5.** Sr isotope values from literature used to build the isoscape of NE Germany.

## REFERENCES


1. D. Pushkina, The Pleistocene easternmost distribution in Eurasia of the species associated with the Eemian *Palaeoloxodon antiquus* assemblage. *Mammal Rev.* **37**, 224–245 (2007).

2. G. Haynes, Late Quaternary proboscidean sites in Africa and Eurasia with possible or probable evidence for hominin involvement. *Quaternary* **5**, 18 (2022).

3. G. E. Konidaris, V. Tourloukis, "Proboscidea-Homo interactions in open-air localities during the Early and Middle Pleistocene of western Eurasia: A palaeontological and archaeological perspective," in *Tuebingen Paleoanthropology Book Series – Contributions in Paleoanthropology Band 1: Human-Elephant Interactions: From Past to Present* (2021).

4. J. Z. Metcalfe, Proboscidean isotopic compositions provide insight into ancient humans and their environments. *Quat. Int.* **443**, 147–159 (2017).

5. T. Weber, The Eemian *Elephas antiquus* finds with artefacts from Lehringenand Gröbern: Are they really Killing Sites? *Anthropologie et Préhistoire* **111**, 177–185 (2000).

6. W. Roebroeks, K. MacDonald, F. Scherjon, C. Bakels, L. Kindler, A. Nikulina, E. Pop, S. Gaudzinski-Windheuser, Landscape modification by Last Interglacial Neanderthals. *Sci. Adv.* **7**, eabj5567 (2021).

7. S. Milano, E. Pop, W. Kuijper, W. Roebroeks, S. Gaudzinski-Windheuser, K. Penkman, L. Kindler, K. Britton, Environmental conditions at the Last Interglacial (Eemian) site Neumark-Nord 2, Germany inferred from stable isotope analysis of freshwater mollusc opercula. *Boreas* **49**, 477–487 (2020).

8. S. Gaudzinski-Windheuser, W. Roebroeks, Eds., *Multidisciplinary studies of the Middle Palaeolithic record from Neumark-Nord (Germany). Volume 1, Halle an der Saale.* (Landesamtes für Denkmalpflege und Archäologie Sachsen-Anhalt – Landesmuseum für Vorgeschichte, 2014).

9. L. Kindler, G. M. Smith, A. García Moreno, S. Gaudzinski-Windeheuser, E. Pop, W. Roebroeks, "The last interglacial (Eemian) lakeland of Neumark-Nord (Saxony-Anhalt, Germany).

Sequencing Neanderthal occupations, assessing subsistence opportunities and prey selection based on estimations of ungulate carrying capacities, biomass production and energy values.” in *Human Behavioural Adaptations to Interglacial Lakeshore Environments. RGZM – Tagungen 37 (Mainz Und Heidelberg [Propylaeum] 2020)*, A. Garcìa-Moreno, *et al.*, Eds. (2020), pp. 67–104. https://doi.org/10.11588/propylaeum.647.

10. F. Marano, M. R. Palombo, Population structure in straight-tusked elephants: A case study from Neumark Nord 1 (late Middle Pleistocene?, Sachsen-Anhalt, Germany). *Boll. Soc. Paleontol. Ital.* **52**, 207–218 (2013).

11. F. Marano, M. R. Palombo, The straight-tusked elephants from Neumark-Nord 1: The state of art. *Alp. Mediterr. Quat.* **24**, 147–149 (2011).

12. S. Gaudzinski-Windheuser, L. Kindler, K. MacDonald, W. Roebroeks, Hunting and processing of straight-tusked elephants 125.000 years ago: Implications for Neanderthal behavior. *Sci. Adv.* **9**, eadd8186 (2023).

13. E. Palkopoulou, M. Lipson, S. Mallick, S. Nielsen, N. Rohland, S. Baleka, E. Karpinski, A. M. Ivancevic, T.-H. To, R. D. Kortschak, J. M. Raison, Z. Qu, T.-J. Chin, K. W. Alt, S. Claesson, L. Dalén, R. D. E. MacPhee, H. Meller, A. L. Roca, O. A. Ryder, D. Heiman, S. Young, M. Breen, C. Williams, B. L. Aken, M. Ruffier, E. Karlsson, J. Johnson, F. Di Palma, J. Alfoldi, D. L. Adelson, T. Mailund, K. Munch, K. Lindblad-Toh, M. Hofreiter, H. Poinar, D. Reich, A comprehensive genomic history of extinct and living elephants. *Proc. Natl. Acad. Sci. U.S.A.* **115**, E2566–E2574 (2018).

14. M. Meyer, E. Palkopoulou, S. Baleka, M. Stiller, K. E. H. Penkman, K. W. Alt, Y. Ishida, D. Mania, S. Mallick, T. Meijer, H. Meller, S. Nagel, B. Nickel, S. Ostritz, N. Rohland, K. Schauer, T. Schüler, A. L. Roca, D. Reich, B. Shapiro, M. Hofreiter, Palaeogenomes of Eurasian straight-tusked elephants challenge the current view of elephant evolution. *eLife* **6**, e25413 (2017).

15. A. Larramendi, Proboscideans: Shoulder height, body mass and shape. *Acta Palaeontol. Pol.* **61**, 537–574 (2016).

16. A. Larramendi, M. R. Palombo, F. Marano, Reconstructing the life appearance of a Pleistocene giant: Size, shape, sexual dimorphism and ontogeny of *Palaeoloxodon antiquus* (Proboscidea: Elephantidae) from Neumark-Nord 1 (Germany). *Boll. Soc. Paleontol. Ital.* **56**, 299–317 (2017).

17. A. Nava, F. Lugli, M. Romandini, F. Badino, D. Evans, A. H. Helbling, G. Oxilia, S. Arrighi, E. Bortolini, D. Delpiano, R. Duches, C. Figus, A. Livraghi, G. Marciani, S. Silvestrini, A. Cipriani, T. Giovanardi, R. Pini, C. Tuniz, F. Bernardini, I. Dori, A. Coppa, E. Cristiani, C. Dean, L. Bondioli, M. Peresani, W. Müller, S. Benazzi, Early life of Neanderthals. *Proc. Natl. Acad. Sci. U.S.A.* **117**, 28719–28726 (2020).

18. S. Gaudzinski-Windheuser, L. Kindler, W. Roebroeks, Widespread evidence for elephant exploitation by Last Interglacial Neanderthals on the North European plain. *Proc. Natl. Acad. Sci. U.S.A.* **120**, e2309427120 (2023).

19. D. Yang, K. Podkovyroff, K. T. Uno, G. J. Bowen, D. P. Fernandez, T. E. Cerling, Strontium isotope mapping of elephant enamel supports an integrated microsampling-modeling workflow to reconstruct herbivore migrations. *Commun. Biol.* **8**, 274 (2025).

20. M. J. Wooller, C. Bataille, P. Druckenmiller, G. M. Erickson, P. Groves, N. Haubenstock, T. Howe, J. Irrgeher, D. Mann, K. Moon, B. A. Potter, T. Prohaska, J. Rasic, J. Reuther, B. Shapiro, K. J. Spaleta, A. D. Willis, Lifetime mobility of an Arctic woolly mammoth. *Science* **373**, 806–808 (2021).

21. N. Kowalik, R. Anczkiewicz, W. Müller, C. Spötl, L. Bondioli, A. Nava, P. Wojtal, J. Wilczyński, M. Koziarska, M. Matyszczak, Revealing seasonal woolly mammoth migration with spatially-resolved trace element, Sr and O isotopic records of molar enamel. *Quat. Sci. Rev.* **306**, 108036 (2023).

22. D. L. Fox, D. C. Fisher, Stable isotope ecology of a Late Miocene Population of *Gomphotherium productus* (Mammalia, Proboscidea) from Port of Entry Pit, Oklahoma, USA. *PALAIOS* **16**, 279–293 (2001).

23. W. Dirks, T. G. Bromage, L. D. Agenbroad, The duration and rate of molar plate formation in *Palaeoloxodon cypriotes* and *Mammuthus columbi* from dental histology. *Quat. Int.* **255**, 79–85 (2012).

24. P. Heddell-Stevens, O. Jöris, K. Britton, T. Matthies, M. Lucas, E. Scott, P. Le Roux, H. Meller, P. Roberts, Multi-isotope reconstruction of Late Pleistocene large-herbivore biogeography and mobility patterns in Central Europe. *Commun. Biol.* **7**, 568 (2024).

25. A. G. Rowe, C. P. Bataille, S. Baleka, E. A. Combs, B. A. Crass, D. C. Fisher, S. Ghosh, C. E. Holmes, K. E. Krasinski, F. Lanoë, T. J. Murchie, H. Poinar, B. Potter, J. T. Rasic, J. Reuther, G. M. Smith, K. J. Spaleta, B. T. Wygal, M. J. Wooller, A female woolly mammoth's lifetime movements end in an ancient Alaskan hunter-gatherer camp. *Sci. Adv.* **10**, eadk0818 (2024).

26. W. J. Bonhof, A. J. E. Pryor, Proboscideans on Parade: A review of the migratory behaviour of elephants, mammoths, and mastodons. *Quat. Sci. Rev.* **277**, 107304 (2022).

27. E. Roditi, H. Bocherens, G. E. Konidaris, A. Athanassiou, V. Tourloukis, P. Karkanas, E. Panagopoulou, K. Harvati, Life-history of *Palaeoloxodon antiquus* reveals Middle Pleistocene glacial refugium in the Megalopolis basin, Greece. *Sci. Rep.* **14**, 1390 (2024).

28. L. Kindler, S. Gaudzinski-Windheuser, F. Scherjon, A. Garcia-Moreno, G. M. Smith, E. Pop, J. D. Speth, W. Roebroeks, Large-scale processing of within-bone nutrients by Neanderthals, 125,000 years ago. *Sci. Adv.* **11**, eadv1257 (2025).

29. R. Anczkiewicz, A. Nava, L. Bondioli, W. Müller, C. Spötl, M. Koziarska, M. Boczkowska, P. Wojtal, J. Wilczyński, High spatial resolution Sr isotope and trace element record of dental enamel mineralization in a woolly mammoth tooth: Implications for paleoecological reconstructions. *Quat. Sci. Rev.* **313**, 108191 (2023).

30. A. Käßner, H. T. Kalapurakkal, B. Huber, M. Tichomirowa, A new water-based $^{87}Sr/^{86}Sr$ isoscape map of Central and NE Germany, with special emphasis on mountainous regions. *Aquat. Geochem.* **29**, 95–125 (2023).

31. D. Pushkina, H. Bocherens, R. Ziegler, Unexpected palaeoecological features of the Middle and Late Pleistocene large herbivores in southwestern Germany revealed by stable isotopic abundances in tooth enamel. *Quat. Int.* **339-340**, 164–178 (2014).

32. N. E. Levin, T. E. Cerling, B. H. Passey, J. M. Harris, J. R. Ehleringer, A stable isotope aridity index for terrestrial environments. *Proc. Natl. Acad. Sci. U.S.A.* **103**, 11201–11205 (2006).

33. S. Pederzani, V. Aldeias, H. L. Dibble, P. Goldberg, J.-J. Hublin, S. Madelaine, S. P. McPherron, D. Sandgathe, T. E. Steele, A. Turq, K. Britton, Reconstructing Late Pleistocene paleoclimate at the scale of human behavior: An example from the Neandertal occupation of La Ferrassie (France). *Sci. Rep.* **11**, 1419 (2021).

34. C. J. Still, J. A. Berry, G. J. Collatz, R. S. DeFries, Global distribution of $C_3$ and $C_4$ vegetation: Carbon cycle implications. *Global Biogeochem. Cycles* **17**, 1006 (2003).

35. T. E. Cerling, J. M. Harris, M. G. Leakey, Browsing and grazing in elephants: The isotope record of modern and fossil proboscideans. *Oecologia* **120**, 364–374 (1999).

36. K. Britton, S. Gaudzinski-Windheuser, W. Roebroeks, L. Kindler, M. P. Richards, Stable isotope analysis of well-preserved 120,000-year-old herbivore bone collagen from the Middle Palaeolithic site of Neumark-Nord 2, Germany reveals niche separation between bovids and equids. *Palaeogeogr. Palaeoclimatol. Palaeoecol.* **333-334**, 168–177 (2012).

37. C. Beirne, T. M. Houslay, P. Morkel, C. J. Clark, M. Fay, J. Okouyi, L. J. T. White, J. R. Poulsen, African forest elephant movements depend on time scale and individual behavior. *Sci. Rep.* **11**, 12634 (2021).

38. E. Berti, B. Rosenbaum, F. Vollrath, Energy landscapes direct the movement preferences of elephants. *J. Anim. Ecol.* **94**, 908–918 (2025).

39. A. Purdon, M. A. Mole, M. J. Chase, R. J. Van Aarde, Partial migration in savanna elephant populations distributed across southern Africa. *Sci. Rep.* **8**, 11331 (2018).

40. K. A. Hoppe, P. L. Koch, R. W. Carlson, S. D. Webb, Tracking mammoths and mastodons: Reconstruction of migratory behavior using strontium isotope ratios. *Geology* **27**, 439 (1999).

41. J. Wall, I. Douglas-Hamilton, F. Vollrath, Elephants avoid costly mountaineering. *Curr. Biol.* **16**, R527–R529 (2006).

42. J. Kioko, A. Horton, M. Libre, J. Vickers, E. Dressel, H. Kasey, P. M. Ndegeya, D. Gadiye, B. Kissui, C. Kiffner, Distribution and abundance of African elephants in Ngorongoro Crater, northern Tanzania. *Afr. Zool.* **55**, 303–310 (2020).

43. L. Zhang, C. Li, C. Yue, H. Luo, X. Li, Q. Yu, J. Li, J. Shen, S. Yang, F. Chen, Habitat suitability evaluation and ecological corridor construction for Asian elephants: The case of Jiangcheng, a new range for elephants in Southwestern China. *Forests* **15**, 1195 (2024).

44. H. Vanleeuwe, Counting elephants in Montane forests: Some sources of error. *Afr. J. Ecol.* **47**, 164–174 (2009).

45. A. Hrynowiecka, R. Stachowicz-Rybka, M. Niska, M. Moskal-del Hoyo, A. Börner, H. Rother, Eemian (MIS 5e) climate oscillations based on palaeobotanical analysis from the Beckentin profile (NE Germany). *Quat. Int.* **605-606**, 38–54 (2021).

46. M. Żarski, H. Winter, M. Kucharska, Palaeoenvironmental and climate changes recorded in the lacustrine sediments of the Eemian Interglacial (MIS 5e) in the Radom Plain (Central Poland). *Quat. Int.* **467**, 147–160 (2018).

47. T. L. Rasmussen, E. Thomsen, A. Kuijpers, S. Wastegård, Late warming and early cooling of the sea surface in the Nordic seas during MIS 5e (Eemian Interglacial). *Quat. Sci. Rev.* **22**, 809–821 (2003).

48. E. Armaroli, F. Lugli, A. Cipriani, T. Tütken, Spatial ecology of moose in Sweden: Combined Sr-O-C isotope analyses of bone and antler. *PLOS ONE* **19**, e0300867 (2024).

49. R. Anczkiewicz, W. Müller, S. Mianowski, M. Dądela, A. Nava, L. Bondioli, M. Matyszczak, A. Jasińska, J. Ostendorf, S. Bakayeva, T. Yanytsky, Comparative high spatial resolution measurements of Sr isotopic composition in bio-apatite using different LA-MC-ICPMS

configurations: Application to faunal (sub)seasonal mobility studies. *J. Anal. At. Spectrom* **40**, 2207–2221 (2025).

50. W. Müller, A. Nava, D. Evans, P. F. Rossi, K. W. Alt, L. Bondioli, Enamel mineralization and compositional time-resolution in human teeth evaluated via histologically-defined LA-ICPMS profiles. *Geochim. Cosmochim. Acta* **255**, 105–126 (2019).

51. C. C. Bakels, "A reconstruction of the vegetation in and around the Neumark-Nord 2 basin, based on a pollen diagram from the key section HP7 supplemented by section HP10." in *Multidisciplinary Studies of the Middle Palaeolithic Record from Neumark-Nord (Germany). Volume 1, Halle an Der Saale.*, S. Gaudzinski-Windheuser, W. Roebroeks, Eds. (Landesamtes für Denkmalpflege und Archäologie Sachsen-Anhalt – Landesmuseum für Vorgeschichte, 2014), pp. 97–107.

52. E. Pop, C. Bakels, Semi-open environmental conditions during phases of hominin occupation at the Eemian Interglacial basin site Neumark-Nord 2 and its wider environment. *Quat. Sci. Rev.* **117**, 72–81 (2015).

53. C. C. Bakels, Non-pollen palynomorphs from the Eemian pool Neumark-Nord 2: Determining water quality and the source of high pollen-percentages of herbaceous taxa. *Rev. Palaeobot. Palynol.* **186**, 58–61 (2012).

54. D. G. Drucker, A. Bridault, K. A. Hobson, E. Szuma, H. Bocherens, Can carbon-13 in large herbivores reflect the canopy effect in temperate and boreal ecosystems? Evidence from modern and ancient ungulates. *Palaeogeogr. Palaeoclimatol. Palaeoecol.* **266**, 69–82 (2008).

55. R. S. Feranec, Stable carbon isotope values reveal evidence of resource partitioning among ungulates from modern $C_3$-dominated ecosystems in North America. *Palaeogeogr. Palaeoclimatol. Palaeoecol.* **252**, 575–585 (2007).

56. E. A. Pearce, F. Mazier, C. W. Davison, O. Baines, S. Czyżewski, R. Fyfe, K. Bińka, S. Boreham, J.-L. de Beaulieu, C. Gao, W. Granoszewski, A. Hrynowiecka, M. Malkiewicz, T. Mighall, B. Noryśkiewicz, I. A. Pidek, J. Strahl, H. Winter, J.-C. Svenning, Beyond the

closed-forest paradigm: Cross-scale vegetation structure in temperate Europe before the late-Quaternary megafauna extinctions. *Earth Hist. Biodivers.* **3**, 100022 (2025).

57. T. E. Cerling, S. A. Andanje, S. A. Blumenthal, F. H. Brown, K. L. Chritz, J. M. Harris, J. A. Hart, F. M. Kirera, P. Kaleme, L. N. Leakey, M. G. Leakey, N. E. Levin, F. K. Manthi, B. H. Passey, K. T. Uno, Dietary changes of large herbivores in the Turkana Basin, Kenya from 4 to 1 Ma. *Proc. Natl. Acad. Sci. U.S.A.* **112**, 11467–11472 (2015).

58. M. Bonafini, M. Pellegrini, P. Ditchfield, A. M. Pollard, Investigation of the 'canopy effect' in the isotope ecology of temperate woodlands. *J. Archaeol. Sci.* **40**, 3926–3935 (2013).

59. N. J. Van Der Merwe, E. Medina, The canopy effect, carbon isotope ratios and foodwebs in Amazonia. *J. Archaeol. Sci.* **18**, 249–259 (1991).

60. L. K. Ayliffe, A. M. Lister, A. R. Chivas, The preservation of glacial-interglacial climatic signatures in the oxygen isotopes of elephant skeletal phosphate. *Palaeogeogr. Palaeoclimatol. Palaeoecol.* **99**, 179–191 (1992).

61. R. Grube, M. R. Palombo, P. Iacumin, A. Di Matteo, "What did the fossil elephants from Neumark-Nord eat?" in *Elefantenreich. Eine Fossilwelt in Europa. Landesamt Für Denkmalpflege Und Archäologie Sachsen-Anhalt, Halle*, H. Meller, Ed. (2010), pp. 253–272.

62. R. Grube, "Pflanzliche Nahrungsreste der fossilen Elefanten und Nashörner aus dem Interglazial von Neumark-Nord." in *Erkenntnisjäger. Festschrift Für Dietrich Mania. Veröffentlichungen Des Landesmuseums Für Vorgeschichte in Halle 57 Halle/Saale*, J. M. Burdukiewicz, L. Fiedler, H. Wolf-Dieter, J. Antje, E. Brühl, Eds. (2003), pp. 221–236. [Plant food remains of the fossil elephants and rhinoceroses from the Neumark-Nord interglacial].

63. J. Saarinen, A. M. Lister, Dental mesowear reflects local vegetation and niche separation in Pleistocene proboscideans from Britain. *J. Quat. Sci.* **31**, 799–808 (2016).

64. F. Rivals, G. M. Semprebon, A. M. Lister, Feeding traits and dietary variation in Pleistocene proboscideans: A tooth microwear review. *Quat. Sci. Rev.* **219**, 145–153 (2019).

65. C. Tsakalidis, G. E. Konidaris, E. Tsoukala, D. S. Kostopoulos, Dietary reconstruction of Pliocene–Pleistocene mammoths and elephants (Proboscidea) from Northern Greece based on dental mesowear analysis. *Quaternary* **8**, 19 (2025).

66. M. R. Palombo, M. L. Filippi, P. Iacumin, A. Longinelli, M. Barbieri, A. Maras, Coupling tooth microwear and stable isotope analyses for palaeodiet reconstruction: The case study of Late Middle Pleistocene Elephas (Palaeoloxodon) antiquus teeth from Central Italy (Rome area). *Quat. Int.* **126-128**, 153–170 (2005).

67. S. Pederzani, K. Britton, Oxygen isotopes in bioarchaeology: Principles and applications, challenges and opportunities. *Earth Sci. Rev.* **188**, 77–107 (2019).

68. K. F. Helmens, The Last Interglacial–Glacial cycle (MIS 5–2) re-examined based on long proxy records from central and northern Europe. *Quat. Sci. Rev.* **86**, 115–143 (2014).

69. N. Kühl, T. Litt, C. Schölzel, A. Hense, Eemian and Early Weichselian temperature and precipitation variability in northern Germany. *Quat. Sci. Rev.* **26**, 3311–3317 (2007).

70. D. R. Green, T. M. Smith, G. M. Green, F. B. Bidlack, P. Tafforeau, A. S. Colman, Quantitative reconstruction of seasonality from stable isotopes in teeth. *Geochim. Cosmochim. Acta* **235**, 483–504 (2018).

71. B. H. Passey, T. E. Cerling, Tooth enamel mineralization in ungulates: Implications for recovering a primary isotopic time-series. *Geochim Cosmochim Acta* **66**, 3225–3234 (2002).

72. K. T. Uno, D. C. Fisher, G. Wittemyer, I. Douglas-Hamilton, N. Carpenter, P. Omondi, T. E. Cerling, Forward and inverse methods for extracting climate and diet information from stable isotope profiles in proboscidean molars. *Quat. Int.* **557**, 92–109 (2020).

73. D. R. Bowling, D. E. Pataki, J. T. Randerson, Carbon isotopes in terrestrial ecosystem pools and $CO_2$ fluxes. *New Phytol.* **178**, 24–40 (2008).

74. L. W. Kuria, D. M. Kimuyu, M. J. Kinyanjui, G. Wittemyer, F. W. Ihwagi, Seasonal variation in the ranging behavior of elephants in the Laikipia-Samburu ecosystem. *Ecol. Evol.* **14**, e70198 (2024).

75. G. Shannon, R. L. Mackey, R. Slotow, Diet selection and seasonal dietary switch of a large sexually dimorphic herbivore. *Acta Oecol.* **46**, 48–55 (2013).

76. S. Ngene, M. M. Okello, J. Mukeka, S. Muya, S. Njumbi, J. Isiche, Home range sizes and space use of African elephants (*Loxodonta africana*) in the Southern Kenya and Northern Tanzania borderland landscape. *Int. J. Biodivers. Conserv.* **9**, 9–26 (2017).

77. K. E. A. Leggett, Home range and seasonal movement of elephants in the Kunene Region, northwestern Namibia. *Afr. Zool.* **41**, 17–36 (2006).

78. C. R. Thouless, Home ranges and social organization of female elephants in northern Kenya. *Afr. J. Ecol.* **34**, 284–297 (1996).

79. E. C. Mills, J. R. Poulsen, J. M. Fay, P. Morkel, C. J. Clark, A. Meier, C. Beirne, L. J. T. White, Forest elephant movement and habitat use in a tropical forest-grassland mosaic in Gabon. *PLOS ONE* **13**, e0199387 (2018).

80. S. Z. Goldenberg, A. K. Turkalo, P. H. Wrege, D. Hedwig, G. Wittemyer, Entry and aggregation at a Central African bai reveal social patterns in the elusive forest elephant *Loxodonta cyclotis*. *Anim. Behav.* **171**, 77–85 (2021).

81. A. K. Turkalo, P. H. Wrege, G. Wittemyer, Long-term monitoring of Dzanga Bai forest elephants: Forest clearing use patterns. *PLOS ONE* **8**, e85154 (2013).

82. G. Bohrer, P. S. Beck, S. M. Ngene, A. K. Skidmore, I. Douglas-Hamilton, Elephant movement closely tracks precipitation-driven vegetation dynamics in a Kenyan forest-savanna landscape. *Mov. Ecol.* **2**, 2 (2014).

83. R. M. Laws, Age criteria for the African elephant: *Loxodonta a. africana*. *Afr. J. Ecol.* **4**, 1–37 (1966).

84. F. Lugli, A. Cipriani, J. Arnaud, M. Arzarello, C. Peretto, S. Benazzi, Suspected limited mobility of a Middle Pleistocene woman from Southern Italy: Strontium isotopes of a human deciduous tooth. *Sci. Rep.* **7**, 8615 (2017).

85. G. J. Parker, J. M. Yip, J. W. Eerkens, M. Salemi, B. Durbin-Johnson, C. Kiesow, R. Haas, J. E. Buikstra, H. Klaus, L. A. Regan, D. M. Rocke, B. S. Phinney, Sex estimation using sexually dimorphic amelogenin protein fragments in human enamel. *J. Archaeol. Sci.* **101**, 169–180 (2019).

86. N. A. Stewart, R. F. Gerlach, R. L. Gowland, K. J. Gron, J. Montgomery, Sex determination of human remains from peptides in tooth enamel. *Proc. Natl. Acad. Sci. U.S.A.* **114**, 13649–13654 (2017).

87. R. Granja, A. C. Araújo, F. Lugli, S. Silvestrini, A. M. Silva, D. Gonçalves, Unbalanced sex-ratio in the Neolithic individuals from the Escoural Cave (Montemor-o-Novo, Portugal) revealed by peptide analysis. *Sci. Rep.* **13**, 19902 (2023).

88. J. Z. Metcalfe, F. J. Longstaffe, G. D. Zazula, Nursing, weaning, and tooth development in woolly mammoths from Old Crow, Yukon, Canada: Implications for Pleistocene extinctions. *Palaeogeogr. Palaeoclimatol. Palaeoecol.* **298**, 257–270 (2010).

89. W. Müller, R. Anczkiewicz, Accuracy of laser-ablation (LA)-MC-ICPMS Sr isotope analysis of (bio)apatite – A problem reassessed. *J. Anal. At. Spectrom* **31**, 259–269 (2016).

90. L. K. Ayliffe, A. R. Chivas, M. G. Leakey, The retention of primary oxygen isotope compositions of fossil elephant skeletal phosphate. *Geochim. Cosmochim. Acta* **58**, 5291–5298 (1994).

91. S. J. Romaniello, M. P. Field, H. B. Smith, G. W. Gordon, M. H. Kim, A. D. Anbar, Fully automated chromatographic purification of Sr and Ca for isotopic analysis. *J. Anal. At. Spectrom* **30**, 1906–1912 (2015).

92. M. Weber, T. Tacail, F. Lugli, M. Clauss, K. Weber, J. Leichliter, D. E. Winkler, R. Mertz-Kraus, T. Tütken, Strontium uptake and intra-population $^{87}Sr/^{86}Sr$ variability of bones and teeth—Controlled feeding experiments with rodents (*Rattus norvegicus*, *Cavia porcellus*). *Front. Ecol. Evol.* **8**, 569940 (2020).

93. J. M. McArthur, R. J. Howarth, T. R. Bailey, Strontium isotope stratigraphy: LOWESS version 3: Best fit to the marine Sr-isotope curve for 0–509 Ma and accompanying look-up table for deriving numerical age. *J. Geol.* **109**, 155–170 (2001).

94. J.-M. Brazier, A.-D. Schmitt, E. Pelt, D. Lemarchand, S. Gangloff, T. Tacail, V. Balter, Determination of radiogenic $^{87}Sr/^{86}Sr$ and stable $\delta^{88/86}Sr_{SRM987}$ isotope values of thirteen mineral, vegetal and animal reference materials by DS-TIMS. *Geostand. Geoanal. Res.* **44**, 331–348 (2020).

95. H. B. Vonhof, S. de Graaf, H. J. Spero, R. Schiebel, S. J. A. Verdegaal, B. Metcalfe, G. H. Haug, High-precision stable isotope analysis of <5 µg $CaCO_3$ samples by continuous-flow mass spectrometry. *Rapid Commun. Mass Spectrom.* **34**, e8878 (2020).

96. C. P. Bataille, I. C. C. von Holstein, J. E. Laffoon, M. Willmes, X. M. Liu, G. R. Davies, A bioavailable strontium isoscape for Western Europe: A machine learning approach. *PLOS ONE* **13**, e0197386 (2018).

97. E. Lightfoot, T. C. O’Connell, On the use of biomineral oxygen isotope data to identify human migrants in the archaeological record: Intra-sample variation, statistical methods and geographical considerations. *PLOS ONE* **11**, e0153850 (2016).

98. C. Ma, H. B. Vander Zanden, M. B. Wunder, G. J. Bowen, ASSIGNR: An R package for isotope-based geographic assignment. *Methods Ecol. Evol.* **11**, 996–1001 (2020).

99. S. J. Sheather, M. C. Jones, A reliable data-based bandwidth selection method for kernel density estimation. *J. R. Stat. Soc. Ser. B* **53**, 683–690 (1991).

100. W. Müller, F. Lugli, J. McCormack, D. Evans, R. Anczkiewicz, L. Bondioli, A. Nava, “Human life histories” in *Treatise on Geochemistry, Third Edition, 8 Volume Set, Vol. 6*, (Elsevier, 2025), pp. 281–328.

101. D. Mania, M. Thomae, T. Litt, T. Weber, *Neumark-Gröbern. Beiträge zur Jagd des mittelpaläolithischen Menschen.* (Deutscher Verlag der Wissenschaften, 1990). [Contributions to the hunting practices of Middle Palaeolithic humans].

102. H. Meller, Ed., *Elefantenreich: Eine Fossilwelt in Europa; Begleitband zur Sonderausstellung im Landesmuseum für Vorgeschichte Halle 26.03.-03.10.2010* (Landesamt für Denkmalpflege und Archäologie Sachsen-Anhalt, Landesmuseum für Vorgeschichte, 2010). [Elephant kingdom: A fossil world in Europe; Companion volume to the special exhibition at the State Museum of Prehistory Halle, 26 March–3 October 2010].

103. D. Mania, M. Altermann, G. Böhme, T. Böttger, E. Brühl, H.-J. Döhle, K. Erd, K. Fischer, R. Fuhrmann, W.-D. Heinrich, R. Grube, P. G. Karelin, J. Koller, K. V. Kremenetski, T. Laurat, J. van der Made, D. H. Mai, U. Mania, R. MusiI, T. Pfeiffer-Deml, E. Pietrzeniuk, T. Schüler, M. Seifert-Eulen, M. Thomae, *Neumark-Nord - Ein interglaziales Ökosystem des mittelpaläolithischen Menschen.* (Veröffentlichungen des Landesamtes für Denkmalpflege und Archäologie Sachsen-Anhalt - Landesmuseum für Vorgeschichte, Band 62, 2010). [Neumark-Nord–An Interglacial ecosystem of Middle Paleolithic humans].

104. M. J. Sier, W. Roebroeks, C. C. Bakels, M. J. Dekkers, E. Brühl, D. De Loecker, S. Gaudzinski-Windheuser, N. Hesse, A. Jagich, L. Kindler, W. J. Kuijper, T. Laurat, H. J. Mücher, K. E. H. Penkman, D. Richter, D. J. J. van Hinsbergen, Direct terrestrial–marine correlation demonstrates surprisingly late onset of the last interglacial in central Europe. *Quatern. Res.* **75**, 213–218 (2011). [Direct terrestrial–marine correlation demonstrates surprisingly late onset of the Last Interglacial in Central Europe].

105. J. Strahl, M. R. Krbetschek, J. Luckert, B. Machalett, S. Meng, E. A. Oches, I. Rappsilber, S. Wansa, L. Zöller, Geologie, Paläontologie und Geochronologie des Eem-Beckens Neumark-Nord 2 und Vergleich mit dem Becken Neumark-Nord 1 (Geiseltal, Sachsen-Anhalt). *E G Quat. Sci. J.* **59**, 120–167 (2011). [Geology, paleontology, and geochronology of the Eem Basin Neumark-Nord 2 and comparison with the Neumark-Nord 1 Basin (Geiseltal, Saxony-Anhalt)].

106. B. Menke, R. Tunni, Das Eeminterglazial und das Weichselfrühglazial von Rederstall/ Dithmarschen und ihre Bedeutung für die mitteleuropäische Jungpleistozän-Gliederung. *Geologisches Jahrbuch. Reihe A, Allgemeine und regionale Geologie BR Deutschland und Nachbargebiete, Tektonik, Stratigraphie, Paläontologie* **A76**, 3–120 (1984). [The Eemian

Interglacial and the Early Weichselian Glacial from Rederstall/Dithmarschen and their significance for the Central European Late Pleistocene stratigraphic subdivision].

107. H. Müller, Pollenanalytische Untersuchungen und Jahresschichtenzählungen an der eem-zeitlichen Kieselgur von Bispingen/Luhe. *Geol. Jahrb.* **A21**, 149–169 (1974). [Pollen-analytical investigations and annual layer counts of the Eemian diatomite from Bispingen/Luhe].

108. S. Lauterbach, F. H. Neumann, R. Tjallingii, A. Brauer, Re-investigation of the Bispingen palaeolake sediment succession (northern Germany) reveals that the Last Interglacial (Eemian) in northern-central Europe lasted at least ~15 000 years. *Boreas* **53**, 243–261 (2024).

109. S. Gaudzinski-Windheuser, E. S. Noack, E. Pop, C. Herbst, J. Pfleging, J. Buchli, A. Jacob, F. Enzmann, L. Kindler, R. Iovita, M. Street, W. Roebroeks, Evidence for close-range hunting by last interglacial Neanderthals. *Nat. Ecol. Evol.* **2**, 1087–1092 (2018).

110. G. Haynes, *Mammoths, mastodonts, and elephants: Biology, behavior, and the fossil record* (Cambridge Univ. Press, 1991).

111. F. J. Stansfield, A novel objective method of estimating the age of mandibles from African elephants (*Loxodonta africana Africana*). *PLOS ONE* **10**, e0124980 (2015).

112. P. C. Lee, S. Sayialel, W. K. Lindsay, C. J. Moss, African elephant age determination from teeth: Validation from known individuals. *Afr. J. Ecol.* **50**, 9–20 (2011).

113. H. Jachmann, Estimating age in African elephants: A revision of Laws' molar evaluation technique. *Afr. J. Ecol.* **26**, 51–56 (1988).

114. G. Haynes, Finding meaning in mammoth age profiles. *Quat. Int.* **443**, 65–78 (2017).

115. J. Hartmann, N. Moosdorf, The new global lithological map database GLiM: A representation of rock properties at the Earth surface. *Geochem. Geophys. Geosyst.* **13**, Q12004 (2012).

116. B. Lehner, G. Grill, Global river hydrography and network routing: Baseline data and new approaches to study the world's large river systems. *Hydrol. Process.* **27**, 2171–2186 (2013).
